# The Potential Role of Probiotics in the Management of Osteoarthritis Pain: Current Status and Future Prospects

**DOI:** 10.1007/s11926-023-01108-7

**Published:** 2023-09-01

**Authors:** Syed Obaidur Rahman, Frédérique Bariguian, Ali Mobasheri

**Affiliations:** 1 Knowledge Centre, WNS Global Services, Gurugram, India; 2 Haleon (Formerly GSK Consumer Healthcare), Route de L’Etraz 2, Case Postale 1279, 1260 Nyon 1, Switzerland; 3 Research Unit of Health Sciences and Technology, Faculty of Medicine, University of Oulu, 90014 Oulu, FI Finland; 4 Department of Regenerative Medicine, State Research Institute Centre for Innovative Medicine, Vilnius, Lithuania; 5 Department of Joint Surgery, First Affiliated Hospital of Sun Yat-Sen University, Guangzhou, China; 6 World Health Organization Collaborating Centre for Public Health Aspects of Musculoskeletal Health and Aging, Liege, Belgium

**Keywords:** Osteoarthritis, Pain, Gut microbiome, Probiotics

## Abstract

**Purpose of Review:**

This narrative review article comprehensively explains the pathophysiology of osteoarthritis (OA) pain perception, how the gut microbiota is correlated with it, possible molecular pathways involved in probiotics-mediated OA pain reduction, limitations in the current research approaches, and future perspectives.

**Recent Findings:**

The initiation and progression of OA, including the development of chronic pain, is intricately associated with activation of the innate immune system and subsequent inflammatory responses. Trauma, lifestyle (e.g., obesity and metabolic disease), and chronic antibiotic treatment can disrupt commensal homeostasis of the human microbiome, thereby affecting intestinal integrity and promoting leakage of bacterial endotoxins and metabolites such as lipopolysaccharides (LPS) into circulation. Increased level of LPS is associated with knee osteophyte severity and joint pain. Both preclinical and clinical studies strongly suggest that probiotics may benefit patients with OA pain through positive gut microbiota modulation and attenuating low-grade inflammation via multiple pathways. Patent data also suggests increased interest in the development of new innovations that involve probiotic use for reducing OA and joint pain.

**Summary:**

Recent data suggest that probiotics are attracting more and more attention for OA pain management. The advancement of knowledge in this area may pave the way for developing different probiotic strains that can be used to support joint health, improve treatment outcomes in OA, and reduce the huge impact of the disease on healthcare systems worldwide.

**Supplementary Information:**

The online version contains supplementary material available at 10.1007/s11926-023-01108-7.

## Introduction

Osteoarthritis (OA) is a chronic, degenerative joint disease that is characterized by progressive deterioration of the entire synovial joint, including articular cartilage, synovium (joint lining), and subchondral bone (bone beneath the cartilage). Symptoms of OA, such as chronic pain and physical limitations, can significantly affect a patient’s individual quality of life. As per the Global Burden of Disease Study 2017, OA leads to the highest years lived with disability rates for nearly three decades among all musculoskeletal disorders [1]. OA has a multifactorial etiology, with different sets of risk factors that can be grouped into two categories, i.e., non-modifiable such as age, ethnicity, sex, genetics, or history of joint trauma or injury, and modifiable such as obesity, occupational health hazard, previous incidence of joint injuries, quadriceps weakness, and joint malalignment [2]. Thus, OA can be considered the phenotypic manifestation of a series of pathways leading to a common end-stage pathology.

Traditionally, OA was believed to be a mechanically driven rather than an inflammatory disease. However, over the years, researchers have discovered that inflammation plays a significant role in cartilage degeneration and impaired repair responses at many stages of the disease [3]. Inflammation plays a central role in OA progression by promoting synovial inflammation, driving chondrocyte-intrinsic catabolic responses, and contributing to the severity of joint symptoms such as pain. The initiation and prolongation of OA are intricately associated with the activation of the innate immune system; thus, inflammation in OA is also characterized as an innate immune response [4]. Studies have reported that the innate inflammatory response during OA creates an environment in joints for increased nociception [5]. Nociceptors are abundant in the joint capsule, ligaments, subchondral bone, and synovium, making it more prone to nociception or heightened pain sensitivity. The nociception has been attributed to increased neuromodulatory mediators such as pro-inflammatory cytokines, chemokines, NGF, neuropeptides, bradykinin, and PGs that can bind to nociceptors [5]. Though nociception during OA originates from peripheral sensitization of nociceptors, it also involves modulation of dorsal root ganglia (DRG) and central sensitization, i.e., prolonged hyperexcitability of the pain circuits in the CNS.

Gut microbiota is a collection of microbial populations, such as bacteria, archaea, and eukarya, responsible for metabolic, immunological, structural, and neurological functions. In a healthy physiological context, pathogenic and symbiotic microbiota coexist in a relative balance (normobiosis) while under pathological stress and/or disease conditions, loss of beneficial microbial input or signal, and an expansion of pathogenic microbes (pathobionts). Thus, diversity and microbial stability are often key indicators of gut microbiota health because of their inverse association with chronic diseases and metabolic dysfunction [6]. Microbial dysbiosis has emerged as a hidden risk factor inducing the production of pro-inflammatory cytokines and bacterial metabolites such as lipopolysaccharides (LPS) [7]. The innate immune system has evolved pattern recognition receptors (PRRs) to recognize bacterial metabolites also categorized as the pathogen-associated molecular pattern (PAMPs). Thus, gut dysbiosis via modulating immune response might increase sensitization of nociceptors and lower pain threshold.

Targeting the gut microbiota through diet and pharmabiotic intervention may represent a new therapeutic strategy for chronic pain management. Probiotics are live or attenuated microorganisms that modify the gut microbiome when administered in an appropriate dose and impart a health benefit to the host [8••]. Probiotic bacterial species can modulate the gut microbiota composition to prevent or improve symptoms of various pathological conditions involving pain disorders. Data from both preclinical and clinical studies have demonstrated that the administration of probiotics such as *Lactobacillus casei* Shirota, *Lactobacillus acidophilus*, *Lactobacillus rhamnosus*, *Lactobacillus casei*, *Clostridium butyricum*, *and Streptococcus thermophiles* could attenuate OA pain (Tables 1 and 2). In addition, probiotics might be effective in modulating OA-associated pain via modulation of multiple signaling pathways, including decreased monocyte chemoattractant protein-1 (MCP-1), C–C chemokine receptor type 2 (CCR2), transient receptor potential cation channel subfamily V member 1 (TRPV1), and calcitonin gene-related peptide (CGRP) expression in the dorsal root ganglion (DRG); downregulation of matrix metalloproteinase (MMP), cyclooxygenase (COX)-2, MCP-1, CCR2, and pro-inflammatory cytokines expression in joint tissues; and increased expression of type II collagen and tissue inhibitor matrix metalloproteinase 1 (TIMP1) (Table 2). Data from clinical trial registries and patent databases show an increased interest in the development of new innovations that involve probiotics as a new therapeutic strategy for managing OA pain. Thus, this review article summarizes preclinical and clinical studies that have demonstrated the effect of probiotics on OA pain. This is the first review article that highlights possible molecular pathways involved in probiotics-mediated OA pain reduction based on published studies. To understand these pathways, the article also discusses how gut microbiota is correlated with chronic low-grade inflammation and innate immune response that drives OA progression and associated pain perception. Finally, the limitations involved in the current research approaches and future perspectives are discussed.

**Table 1:** Clinical studies demonstrating role of probiotics in OA pain

Probiotic strain(s)	Study design*	Population	Dose; duration	Result		Other remarks
				Pain scale/score	Other markers studied	
*Lactobacillus casei* Shirota [9]	Randomized controlled trial	461 patients with knee OA	461; 12 × 10^9^ CFU once daily; 6 months	↓ VAS and WOMAC score	↓ Serum hs-CRP	• Strong linear correlations between serum hs-CRP levels, WOMAC, and VAS scores were observed
*Streptococcus thermophiles* [10••]	Randomized controlled trial	67 patients with knee OA	20 × 10^8^ CFU once daily; 12 weeks	↔ WOMAC scores	↑ Serum sCTX-II and sCRP	• Pain, stiffness and function neither improved nor worsened after 12 weeks of probiotic treatment • Changes in serum sCTX-II and sCRP indicated a potential for alleviation of OA and the prevention of joint degeneration
*Lactobacillus rhamnosus* + *Saccharomyces cerevisiae* + *Bifidobacterium animalis* ssp. *lactis* [11••]	N-of-1 trial	67-year-old female with a history of OA	20 × 10^9^ CFU once daily; 32 weeks (3 blocks of 10-week duration each with a 2-week follow-up)	↓ VAS score	↓ PSFS score ↔ SCFA level ↓β-Glucuronidase levels ↓*Bifidobacteria* and *E.coli*	• The reduction in pain scores associated with the probiotic intervention was small but clinically significant for the patient

^*^All studies were placebo-controlled

*CFU*, colony-forming units; *hs-CRP*, high-sensitivity C-reactive protein; *PSFS*, patient-specific functional scale; *SCFA*, short-chain fatty acids; *sCRP*, serum C-reactive protein; *sCTX-II*, serum collagen type II C-telopeptide; *VAS*, visual analog scale; *WOMAC*, Western Ontario and McMaster universities

*↑*, increase; *↓*, decrease; ↔ , no change

**Table 2:** Preclinical studies demonstrating the role of probiotics in OA pain

Probiotic strain(s)	Dose; duration	Results						
		Pain behavior	Dorsal root ganglion	Synovium	Cartilage	Serum	Intestine	Microbiota
Monosodium iodoacetate (MIA)–induced OA								
*Lactobacillus acidophilus* [12]	NA; 15 days	↑ PWL; PWT; WBC	↓ TRPV-1 expression; CGRP release	↓ TNF-α; IL-6; MMP3 ↑ IL-10; TIMP3	↓ OARSI and total Mankin Score	−	−	−
*Lactobacillus acidophilus* [13]	125 mg/ml (2 × 10^11^ CFU/ml)/day; 24 days	↑ PWL; WBC	↓ TRPV-1 expression; CGRP release	↓ TNF-α; IL-1β; MCP-1 MMP-13	↑ GPR43 + cells	−	↓ loss of epithelium; goblet cells depletion; crypt damage; TNF-α; IL-1β; MCP-1 ↑ TJP expression	↓ *Bacteriodes* ↑ F/B ratio; *Bifidobacterium; Faecalibacterium prausnitzii*
*Lactobacillus rhamnosus* [14]	NA; 28 days	↑ PWL; PWT; WBC	↓ MCP-1 and CCR2 ↑ GABA release and PPAR-γ expression	↓ MCP-1 and CCR2 ↑ IL-10; TIMP3	↓ OARSI and total Mankin score	−	↓ MCP-1; CCR2; IL-6 ↑ IL-10	−
*Lactobacillus casei* [15]	2 × 10^10^ CFU/kg/day; 8 weeks	↑ PWT	−	↓ COX-2; TNF-α; IL-6; IL-1β; MMP1,3 & 13 ↑ TIMP1	↓ COX-2; TNF-α; IL-6; IL-1β; MMP1,3 & 13 ↑ TIMP1	−	−	−
*Clostridium butyricum* [16]	10^10^ CFU/day; 2 weeks (pre-treatment) and 4 weeks (post-treatment)	↑ WBC	−	−	↓ TIMP1 and 3; MMP2,3,9 and 13; OARSI score	**↓** COX-2; LTB4; IFN-γ and IL-6	−	−
Anterior cruciate ligament transection (ACLT)–induced OA								
*Clostridium butyricum* [17]	100 mg/kg/day (5.5 × 10^7^ CFU/g); 6 weeks	↑ WBC	−	**↓** IL-1β; TNF-α	**↓** OARSI scores; cartilage degeneration scores; synovial tissue inflammation scores; IL-1β; TNF-α	−	−	−
*Lactobacillus plantarum* [18]	100 mg/kg (5 × 10^10^ CFU/kg)/day; 6 weeks	↑ WBC	−	**↓** IL-1β; TNF-α	**↓** OARSI scores; cartilage degeneration scores; synovial tissue inflammation scores; IL-1β; TNF-α	−	−	−
*Streptococcus thermophilus* [19]	5 × 10^9^, 5 × 10^10^, and 5 × 10^11^ CFU/kg/day; 24 weeks	↑ PWT; PWD	−	↓Synovial score	↓ OARSI score ↑ Type II collagen	−	−	−
Partial medial meniscectomy (PMM)–induced OA								
*Lactobacillus acidophilus* [20]	3 × 10^9^ CFU/200 µL twice/week; 11 weeks	↑ PWT	**↓** TRPV1 expression	−	**↓** OARSI scores; MMP13; RUNX2	−	**↓** IL-1β; TNF-α; NF-κB ↑ IL-10	↑ Lachnospiraceae

*CCR2*, C–C chemokine receptor type 2; *CFU*, colony-forming unit; *CGRP*, calcitonin gene-related peptide; *COL2A1*, alpha-1 chain of type II collagen; *COX-2*, cyclooxygenase 2; *DRG*, dorsal root ganglion; *GABA*, gamma-aminobutyric acid; *IFN-γ*, interferon-gamma; *IL*, interleukin; *LTB4*, leukotriene B4; *MCP-1*, monocyte chemoattractant protein-1; *MMP*, matrix metalloproteinase; *NF-κB*, nuclear factor kappa B; *OARSI*, osteoarthritis research society international; *PPAR-γ*, peroxisome proliferator-activated receptor gamma; *PWD*, paw weight difference; *PWL*, paw withdrawal latency; *PWT*, paw withdrawal threshold; *SOX9*, SRY-box transcription factor 9; *TIMP*, tissue inhibitor; *TJP*, tight junction protein; *TRPV1*, transient receptor potential cation channel subfamily V member 1; *WBC*, weight bearing capacity

↑, increase; ↓, decrease; ↔ , no change

## Chronic Low-grade Inflammation Is the Key Mediator of OA Progression

OA was traditionally considered the sole consequence of any process leading to increased pressure on one particular joint or fragility of the cartilage extracellular matrix (ECM). However, the advancement in molecular biology and the discovery of cytokines and the role of PG in the pathogenesis of OA have led to the increased acceptance of an “inflammatory” theory. The combination of sensitive imaging modalities and direct arthroscopic visualization has suggested the involvement of synovial inflammation in both early OA and advanced-stage OA that further led to the development and progression of OA. The presence of synovitis often predates the development of radiographic damage in OA, and mononuclear cell infiltration and overexpression of inflammatory mediators in the synovium are prominent in early OA [21, 22]. It should be noted that the critical role of synovitis includes the involvement of cartilage and the chondrocyte in the pathogenesis of either early or late OA. Cartilage breakdown products in synovial fluid and microfissures in articular cartilage are present long before any degeneration can be noted using current MRI technology or gross arthroscopic visualization [23]. Early cartilage degradation events may play a driving role in the development of inflammation within the OA joint, specifically the OA synovium. Thus, OA can be viewed as a vicious, self-perpetuating cycle of local tissue damage, inflammation, and maladaptive repair responses, leading to chronic low-grade inflammation.

## OA Progression, Activation of the Innate Immune System, and Subsequent Inflammatory Responses

The inflammation observed in OA has been reported to be a consequence of our immune response, primarily innate and, to a lesser degree, adaptive [24]. PRRs, such as Toll-like receptors (TLRs) or nucleotide-binding oligomerization domain (NOD)–like receptors (NLRs) are important for sensing components of but not limited to, microbial cells, such as lipopeptides, peptidoglycans, glycolipids, and lipopolysaccharides (LPS),

Some PRRs can also detect host-derived danger signals generated when tissues are damaged. One such group of molecules known to generate these signals is damage-associated molecular patterns (DAMPs). DAMPs signal to the immune system a state of stress requiring a protective response to either combat infection or initiate repair processes. DAMPs found to be involved in the progression of OA can be divided into four categories, i.e., cartilage ECM components (fibronectin, hyaluronan, biglycan, and tenascin C), plasma proteins (Gc-globulin, α1-microglobulin, and α2-macroglobulin), intracellular alarmins (high mobility group box 1 and S100), and crystals (basic calcium phosphate, calcium pyrophosphate dihydrate, uric acid) [22]. Because the list of innate DAMPs is long and rapidly growing, a complete discussion is beyond the scope of this review.

OA is characterized by progressive tissue degradation that mainly affects the articular cartilage but is also evident in the surrounding tissues. Multiple cell types within the joint express PRRs, including macrophages, fibroblasts, and chondrocytes [24, 25]. As a result, in the joints of patients with OA, these damaged tissues might generate multiple DAMPs and alarmins, which after binding to their respective receptors, would promote cellular innate immune responses. Studies have shown that the expression of PRRs such as TLR2, TLR4, receptor for advanced glycation end products (RAGE), and NLR family pyrin domain containing 3 (NLRP3) in cartilage is increased throughout the development of OA [24]. Along with microbe-associated molecular patterns (MAMPs), TLR4 also senses more DAMPs than any other known PRR [26, 27]. Moreover, the transit of plasma proteins into the OA synovial compartment induces TLR4-mediated inflammatory responses in macrophages, which are important mediators of synovial inflammation in OA. Soluble inflammatory factors including cytokines, chemokines, adipokines, neuropeptides, and lipid inflammatory mediators have been implicated in OA pathogenesis [22].

**Fig. 1:**
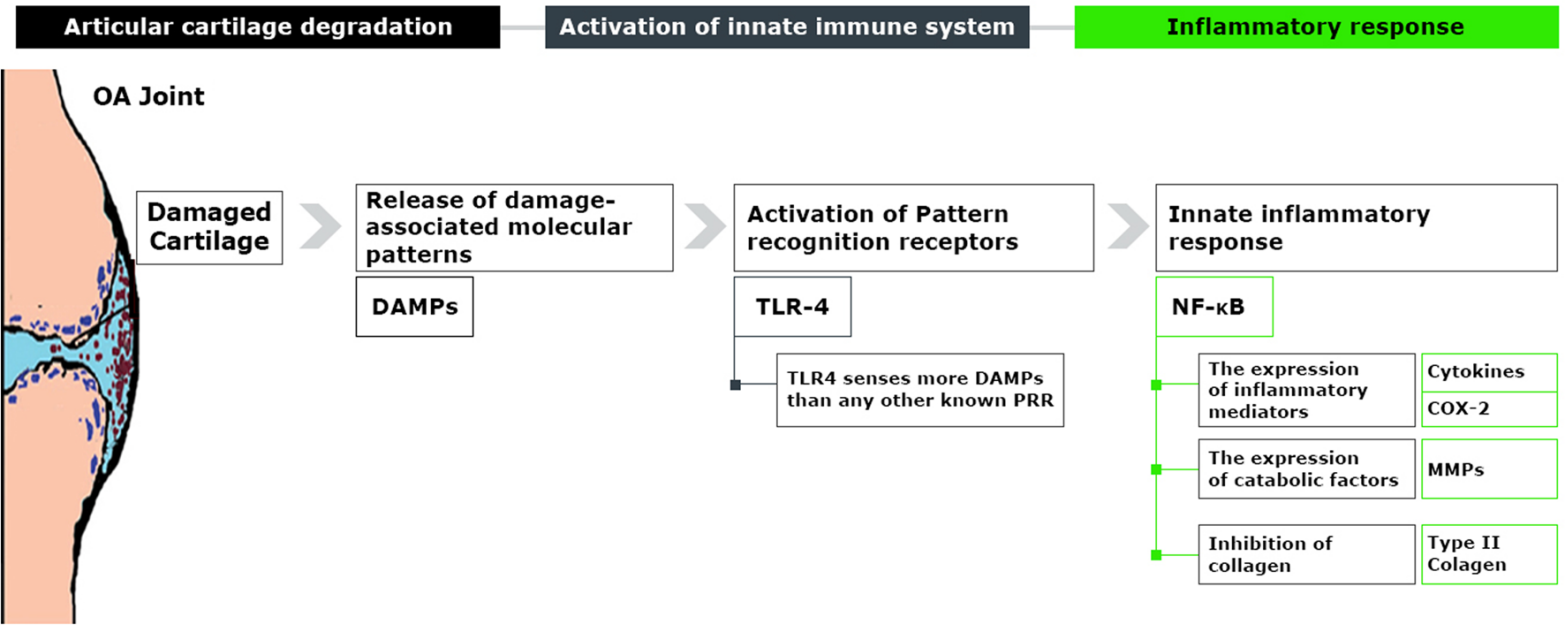
The role of innate immune system in the initiation and prolongation of OA

Nuclear factor-κB (NF-κB) has been recognized as the master orchestrator of TLR-induced responses, as all TLR signals converge on NF-κB [28]. The NF-κB pathway is a regulator for inflammatory processes in chondrocytes. It increases the expression of MMPs, nitrous oxide synthase (NOS), COX-2, and interleukin-1 (IL-1), and inhibition of type II collagen and aggrecan core protein expression, which can cause apoptosis, a shift in cell phenotype, and overexpression of pro-inflammatory genes that ultimately contribute to further destruction of cartilage ECM (Fig. 1) [29]. Benito et al. found a positive correlation between NF-κB1 and increased COX-2 expression in knee joint synovial tissues retrieved from patients with early-phase OA [21]. Liu et al. investigated the expression of inflammatory factors during OA progression [30]. They found that expression of TLR-2, NF-κB, and MMP-13 is upregulated with the increase in the degree of OA lesions, indicating that the TLR-2/NF-κB signaling pathway can contribute to OA development. Similarly, Ostojic et al. discussed the role of macrophages in OA progression through the NF-κB-mediated production of inflammatory factors (i.e., iNOS and MMP-9) in the intima, except in advanced OA, where leukocytes could have a dominant role through NF-κB production in subintima [31]. The high expression of NF-κB in synovial macrophages was highlighted as direct evidence of the involvement of macrophages in the pathogenesis of knee OA.

## In Addition to Local Inflammation in the Joint, Systemic Inflammation Might Also Have an Important Role in OA Pathogenesis

Metabolic abnormalities such as obesity are known to predispose individuals to OA, possibly not only by increasing the mechanical load on joints but also by causing chronic, systemic inflammation through inflammatory mediators (such as adipokines and other pro-inflammatory cytokines) that are produced by adipose tissue and released into the bloodstream [32]. It is possible that the systemic inflammation associated with chronic inflammatory states, such as obesity or certain chronic diseases, promotes local inflammation in joints that ultimately results in OA.

Several studies support the observation that systemic inflammation is associated with OA. These include key epidemiologic analyses demonstrating that serum levels of C-reactive protein (CRP) are strongly associated with the presence and progression of knee OA [33]. Snelling et al. demonstrated a high level of synovial fluid-high sensitivity C-reactive protein (SF-hs-CRP) in knee OA. They suggested it as an indicator of synovial inflammation and ongoing inflammatory process [34]. A positive correlation between levels of serum CRP and histologic evidence of synovitis and synovial fluid interleukin-6 (IL-6) at the time of joint replacement has also been demonstrated. These observations strongly suggest that the systemic inflammation observed in OA is at least partially reflective of local synovial inflammation [35].

## The Complex Interplay Between the Gastrointestinal Microbiome, Its Products, and the Immune System Triggers the Role of Low-grade Inflammation in OA

The term “gut joint axis” was coined to explain the presence of an interlinked pathway that involves the gastrointestinal microbiome, immune responses, and joint pathophysiology. Over the years, research has focused on understating the role of this axis in the etiology and pathogenesis of OA. The correlation between the altered bacterial composition of the gut microbiome and OA severity suggests systemic implications for the disruption of gut permeability. Specific groups of bacteria such as Firmicutes, Bacteroidetes, and Streptococcus have been implicated in the pathogenesis of gut dysbiosis associated with OA. Several preclinical studies have established that obesity and high-sugar and high-fat diets modulate gut microbiome composition and result in increased Firmicutes/Bacteroidetes (F/B) phyla ratio and increased permeability [36•]. An increase in the F/B ratio resulted in dysregulation of the delicate balance between pro-inflammatory markers (IL-1β, IL-6, TNFα, IL-12, IL-18) and thus contributing towards a pro-inflammatory state.

Substantial evidence from preclinical and clinical studies suggests that bacterial products can leak across the gut epithelium and reach many organs through the systemic circulation and thus cause inflammation especially in certain comorbid conditions, such as obesity [37]. Since then, connections have been hypothesized and proven between the microbiome and a variety of different diseases, such as inflammatory bowel disease [38], type 2 diabetes [39], and atherosclerosis [40]. Enrico et al. reviewed a noticeable connection between the gut and musculoskeletal health and discussed the crucial role of the gut microbiome in mediating systemic inflammation [41•]. The authors described the gut microbiome as an endocrine organ functionally based upon the fact that the gut microbiome produces numerous chemicals (frequently defines as hormones in review articles) that act via the bloodstream to distal sites in the human body.

Given the intimate interplay between gut microbiota-derived metabolites and the host immune system, it is not surprising that some microbial components have been linked to autoimmune responses via the activation of innate immunity. Components of the microbiome affect the immune composition of the gut through the maturation and differentiation of immune cells. The microbiome produces microbial components and metabolites recognized by the innate immune system. This induces constitutive signaling, much of which occurs via toll-like receptors. This signaling produces factors that induce a physiologic level of inflammation. One such component of the outer membrane of gram-negative bacteria is LPS, which elicits cartilage and synovium inflammatory pathways. The bacterially produced pro-inflammatory metabolites, such as LPS, make their way from the “leaky gut” to the systemic circulation and induce systemic inflammation [42]. LPS is now known to contribute to systemic inflammation with several clinical conditions, including cardio-metabolic dysfunction, acceleration of atherosclerosis, and diabetes. Huang et al. found that increased LPS and LPS-binding were associated with knee osteophyte severity and abundance of activated macrophages in the synovium. Also, monitoring circulating LPS concentrations could provide a new means to diagnose and treat specific phenotypes of OA [43]. LPS is a classical activator of the innate immune system that activates immune cells, such as macrophages and neutrophils, in the host by binding to the TLR4 complex. Because of its pathophysiological properties, LPS has been used to induce arthritis in conjunction with collagen in animal models [44]. LPS and DAMPs synergistically activate an innate immune-driven inflammatory response.

## Gut Dysbiosis Occurs Either Due to Lifestyle Modification (e.g., Obesity and Metabolic Disease) or Chronic Antibiotic Treatment, Significantly Affecting Gut Permeability

The most accredited linking factor between dysbiosis and OA may be represented by the common appearance of chronic systemic inflammation, supporting the existence of a new OA phenotype indicated as metabolic OA. Obesity, one of the most well-known risk factors for OA, increases the mechanical stress on the tibiofemoral cartilage and leads to a higher prevalence of OA in non-weight-bearing areas owing to a link between obesity and metabolic syndrome. Obesity involves inflammatory cytokines or adipokines release induced by immune response [32]. Indeed, obesity is associated with impaired gut mucosa and microbiome translocation [45]. High-fat diet modulates tight junctions, their expression, and distribution directly through dietary fats or indirectly via increased cytokines release [46]. Moreover, microbes are spatially redistributed in the intestine, mainly occupying intervillous/cryptal spaces. Due to such changes, progressively, innate immune receptors in the gut are activated by microbial products and stimulate pro-inflammatory mediators’ production. Pro-inflammatory cytokines, in turn, dysregulate tight junction formation creating a vicious cycle. Metabolic endotoxemia, caused by impaired gastric mucosa and inflammation, may contribute to the onset and progression of OA in obese patients [47]. This may account for the association between obesity and OA at non-weight-bearing joints, which biomechanical factors cannot explain. In OA, macrophage activation has been implicated as one of the predominant immune responses responsible for increased pain and inflammation [48]. Macrophages can be activated by the presence of bacterial products systemically or in their resident tissue. Specifically, LPS, produced by many bacteria including *Streptococcus*, can bind TLR4 expressed by macrophages [49]. This can induce pro-inflammatory mediators and lead to increased intestinal permeability. Increased intestinal permeability allows greater passage of bacteria, bacterial fragments, and pro-inflammatory mediators into the systemic circulation. Due to the elevation of LPS levels in association with obesity and metabolic syndrome, which are highly relevant risks factors for OA, it is possible to speculate on the involvement of the gut microbiota in OA, at least by LPS-induced inflammation, metabolic endotoxemia, macrophage activation, and consequent joint damage.

Although gut microbiota dysbiosis has been studied in the context of OA, the effect of antibiotic-induced gut flora dysbiosis on OA still needs to be mechanistically explored. Several recent investigations have explored the effect of antibiotic treatment on OA progression, and the results are described in this section. Reduction of gut microbiome through antibiotic treatment has also demonstrated reduced articular cartilage structure damage in mice subjected to joint compression loading [50]. The most likely explanation for the reduced cartilage damage in the antibiotic-treated mice is alterations in the gut microbiome. In a mouse OA model, antibiotic-induced destabilization of the medial meniscus (DMM) and gut microbiome dysbiosis reduced LPS serum levels. In addition, the inflammatory response, such as suppression of the levels of tumor necrosis factor-α (TNF-α) and interleukin-6 (IL-6), decreased MMP-13 expression and thus led to improvement of OA after joint injury. Moreover, osteophyte scores and trabecular thickness were increased significantly in antibiotic-induced male mice compared with female mice [51••]. Similarly, oral doxycycline has been found to reduce OA joint pathology in humans and animals [52, 53]. The other most important and neglected component in the studies is the long-term impact of antibiotic administration on the host’s healthy microbiome. Regular consumption of antibiotics to treat common bacterial infections can cause antibiotic‐induced changes in microbial composition and can harm host health, including reduced microbial diversity and changes in functional attributes of the microbiota.

## Association Between the Gut Microbiome and OA Pain

### Nociception or Heightened Pain Sensitivity During OA Has Been Attributed to the Innate Inflammatory Response

Joint pain can be categorized as nociceptive pain, which occurs in response to immediate tissue damage, and serves as an early-warning protective response; and neuropathic pain, which develops from damage to the somatosensory nervous system that does not diminish as the body heals. The development of neuropathic pain is not within the scope of this article.

**Fig. 2:**
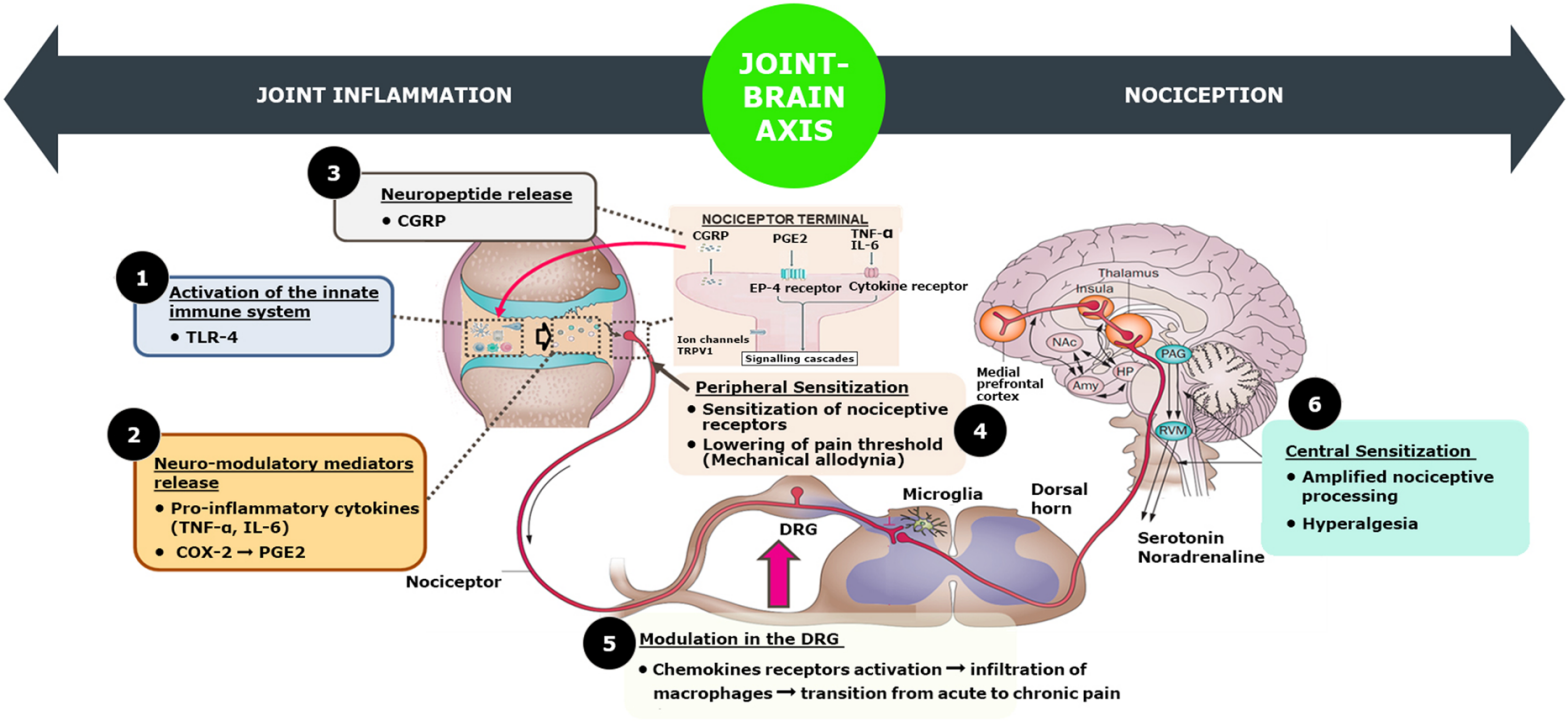
Joint brain axis illustrating both peripheral and central processes leading to the OA pain experience

The joint is a densely innervated organ. Its sensory innervation is geared predominantly towards proprioception and nociception, indicating how vital positioning sense and awareness of potentially harmful movement are to proper joint function. The sensory innervation comprises pain receptors, called nociceptors (from the Latin word “noxa,” which means damage and receptors), sending the hurt signal to the spinal cord and then to the CNS. Nociceptors are pseudo-unipolar cells with an axonal stalk that extends from the cell body in the DRG and splits into two terminals. The peripheral terminal innervates peripheral tissues, and the central terminal extends to the dorsal horn of the spinal cord. Thus, nociceptors can receive and send signals at both terminals. In addition, nociceptors are abundant in the joint capsule, ligaments, periosteum, menisci, subchondral bone, and synovium, making it more prone to nociception. This heightened pain sensitivity is attributed to a number of factors outlined below and in Fig. 2.

#### Peripheral Sensitization

Nociceptors can be activated by neuromodulatory mediators such as pro-inflammatory cytokines, chemokines, NGF, neuropeptides, bradykinin (BK), and PGis, which can enhance nociceptors sensitivity [5]. The noxious stimulus is then transduced into an electrical signal generating an action potential along the primary afferent neuron. In normal joint conditions, there is a high threshold stimulus of a mechanical, thermal, or chemical nature. But during OA, the sensitivity of the nociceptor increases, and even nociception occurs even at a lower threshold and leads to physical limitations. Active nociceptors release neurotransmitters, i.e., substance P (SP), CGRP, and vasoactive intestinal peptide (VIP), from peripheral terminals [5]. The release of these neurotransmitters may be associated with vasodilation inducing extracellular leakage of fluid and proteins. Overall, these inflammatory conditions result in immune system activation that, in a vicious circle, enhances inflammation and ultimately leads to pain perception. Several target receptors and ion channels on peripheral terminals and along axons of primary afferent neurons are involved in pain stimulus transduction. Sensitization of nociceptor leads to decreased activation threshold for neuronal ion channels, such as the TRPV1, thus contributing to mechanical hyperalgesia (increased sensitivity to noxious stimuli) and allodynia (the interpretation of non-noxious stimuli as painful) [5].

#### Modulation in the DR

Voltage-gated ion channels (i.e., voltage-gated sodium: Nav 1.7, Nav 1.8, potassium, or voltage-dependent calcium channels) convey nociceptor signals to electrical signals to be propagated through the primary afferent neuron to the synapses located in the dorsal horn of the spinal cord [54]. Neuronal cell bodies in the DRG reside alongside small satellite glial cells and macrophages, and their interactions may promote the transition from acute to chronic pain. As the disease progresses, the expression of the MCP1 and its receptor, CCR2, is increased, followed by infiltration of macrophages into the DRG and by the onset of pain behaviors [55].

#### Central Sensitization

Central termini of afferent neurons enter the dorsal horn of the spinal cord and make their first synapse with interneurons or projection neurons. Continued nociceptor input can lead to prolonged hyperexcitability of pain circuits in the CNS, a phenomenon known as central sensitization. Cellular processes involved are increased neuronal membrane excitability, synaptic facilitation, and disinhibition. Overall, central sensitization is the result of the tremendous plasticity of the CNS. It leads to increased spontaneous neuronal activity, reduced activation thresholds, and expansion of the receptive field. It is manifested as hyperalgesia (increased sensitivity to noxious stimuli) and allodynia (the interpretation of non-noxious stimuli as painful), even in areas outside the initial trigger zone. Mounting evidence suggests that central sensitization phenomena are integral to OA pain [56]. Furthermore, abnormalities in somatosensory perception are reversible after successful surgery or joint replacement, as reported for hip OA and knee OA [57]. This reversibility further underlines the plasticity of the CNS and, mechanistically, implies that central sensitization is maintained by peripheral input from the joint.

## Gastrointestinal Microbiota Can Directly or Indirectly Modulate Mechanisms Underlying Chronic Pain Through Multiple Gut Microbiota-Derived Mediators

**Fig. 3:**
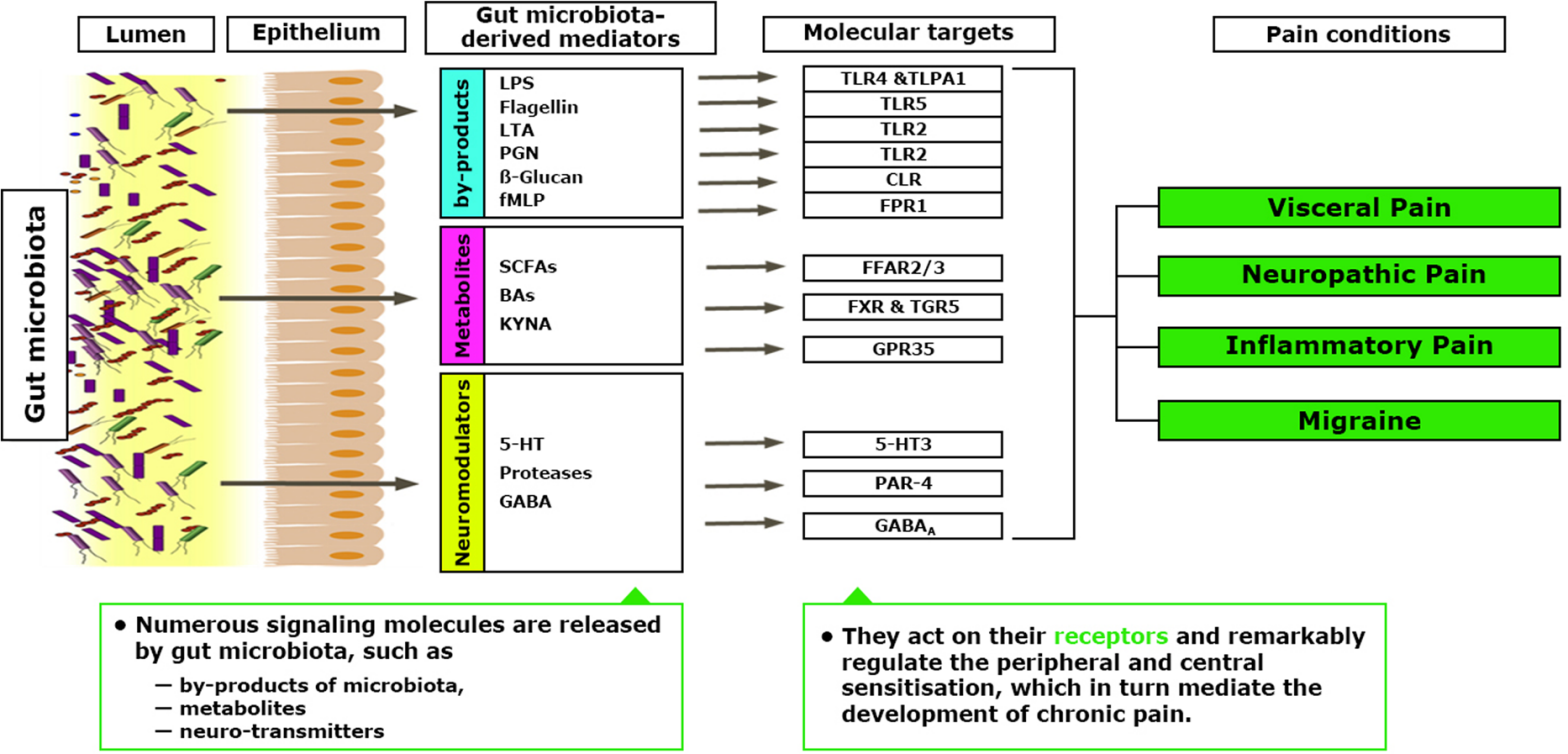
Effect of gastrointestinal microbiota on peripheral sensitization triggering chronic pain

The role of the gut microbiota in visceral or abdominal pain is well established. However, their influence on chronic pain, such as inflammatory pain, neuropathic pain, headache, and opioid tolerance, has only been recently recognized [8••]. Gastrointestinal microbiota can directly or indirectly modulate peripheral sensitization underlying chronic pain through multiple gut microbiota-derived mediators, including microbial by-products, metabolites, and neurotransmitters or neuromodulators release (Fig. 3) [58••]. In addition, the gut microbiota can modulate dorsal root ganglia neuronal excitability and regulate neuroinflammation in the peripheral and central nervous systems under chronic pain conditions.

Pathogen-associated molecular patterns (PAMPs) act on immune cells to release pro-inflammatory cytokines and chemokines indirectly activated or sensitized primary sensory neurons in DRGs. On the other hand, primary sensory neurons in DRGs can be directly activated or sensitized by PAMPs. For example, LPS can bind to TLR4 to induce activation and sensitization of nociceptive neurons in DRGs, partially via a TRPV1-mediated mechanism [59, 60]. Moreover, LPS directly activates the transient receptor potential ankyrin 1 (TRPA1) channel and induces the release of CGRP, calcium flux, and action potentials in nociceptive sensory neurons through a TLR4-independent manner [61]. Thus, bacteria can produce pain by directly activating primary sensory neurons, which reveals an unsuspected role of primary sensory neurons in microbiome-host interaction. Therefore, PAMPs derived from gut microbiota may contribute to peripheral sensitization by directly acting on primary nociceptive neurons or indirectly acting on immune cells to induce neuronal hyperexcitability, leading to peripheral sensitization. In addition, activation of glia (e.g., microglia and astrocytes) can produce pro-inflammatory cytokines or chemokines, such as TNF-a, IL-1β, and CXCL1, and can result in elevated glutamatergic synaptic neurotransmission, decreased GABAergic synaptic neurotransmission, or both [62–64]. Both effects contribute to the development of central sensitization, leading to pain hypersensitivity [65]. Notably, gut microbiota plays a pivotal role in regulating microglia’s maturation, morphology, and immunological function [66, 67]. However, further investigation is needed to determine whether the neuroinflammation-mediated central sensitization underlying chronic pain can be directly or indirectly regulated by the gut microbiota.

## Association Between the Gut Microbiome and OA-Related Pain

Several studies indicate that gut microbiota may play an important role in inflammatory pain [68•]. Amaral and team found that the inflammatory pain induced by carrageenan, lipopolysaccharide (LPS), TNF-a, IL-1β, and chemokine CXCL1 was reduced in germ-free mice compared with conventional mice [69]. The reduced pain hypersensitivity of the germ-free mice was significantly aggravated after being transplanted with the stool of conventional mice. Notably, decreased pain hypersensitivity in germ-free mice was associated with enhanced expression of interleukin 10 (IL-10) upon stimulation and can be reversed by an anti-IL-10 neutralizing antibody [69]. Recently, Yan and Kentner demonstrated that neonatally LPS-challenged rats showed increased mechanical hypersensitivity during adolescence, and the administration of a broad-spectrum antibiotic cocktail attenuated this mechanical hypersensitivity [70]. Injection of monosodium urate monohydrate (MSU) crystals caused joint inflammation, hypernociception, and production of IL-1β and CXCL1, which were substantially decreased in germ-free mice, mice treated with antibiotics, and free fatty acid receptor 2 (FFAR2 or GPR43)–deficient mice, suggesting commensal microbiota play a critical role in gout-induced acute inflammation and pain [71]. Thus, this suggests that the gut microbiota may play a key role in the direct or indirect regulation of pain perception under chronic pain conditions.

More high-quality clinical studies are needed to address the potential role of the gut microbiome in OA-related pain. The paucity of clinical studies along with the disparity of the techniques used so far makes it impossible to draw firm conclusions on the topic. However, there are some interesting trends in the literature. Romero et al. found three studies that supported a relationship, specifically a correlation between the levels of certain taxa or microbial products, namely LPS, and the inflammatory landscape and severity of OA symptoms, including knee WOMAC pain [72••]. The major microbial taxa hypothesized to be involved *Clostridium* and *Streptococcus* species, with the former having been shown to promote T helper 17 (Th17) cells and drive arthritis and the latter postulated to lead to increased knee pain through activation of local or systemic macrophages [73, 74]. These assumptions are consistent with the previous study by Huang et al., which associates the presence of LPS and LPS-binding protein in both the serum and synovial fluid of OA patients with that of activated macrophages in the knee, OA severity, and joint symptoms, mainly pain [75]. Dunn et al. revealed a microbial DNA signature in the knee and hip cartilage samples from OA patients [76••]. The study also demonstrated an increase in gram-negative constituents such as LPS in human OA cartilage.

It is reasonable to speculate that a dysbiotic gut microbiome contributes to eliciting a local and systemic inflammatory state, also through the leakage of microbial products or metabolites across an impaired epithelial barrier, ultimately inducing or exacerbating OA-related pain. Favazzo et al. reviewed the gut microbiome-joint connection and highlighted (a) correlations between serum levels of bacterial metabolites and joint degeneration; (b) microbial community shifts induced by antibiotics, a germ-free environment, or high-fat diet; (c) dietary supplementation with nutraceuticals that are joint protective may exert their influence via shifts in the gut microbiome [77•].

**Fig. 4:**
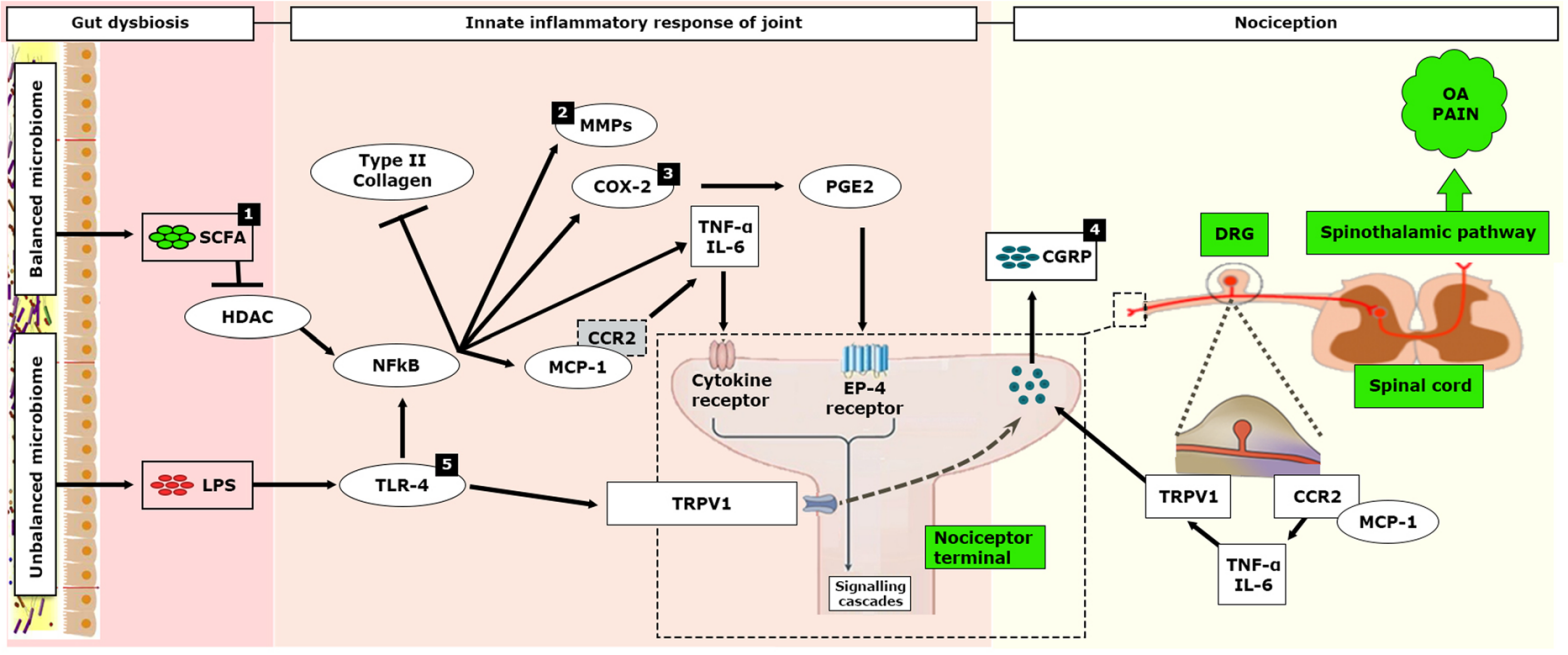
Association between the gut microbiome and OA pain: proposed mechanisms and molecular pathways. ^1^ SCFA inhibits NF-κB and pro-inflammatory cytokines release by inhibiting histone deacetylation (HDAc). ^2^ The accumulation of MMPs in osteoarthritic cartilage may have a role in the biphasic progression of OA-related pain. ^3^ Pro-inflammatory cytokines, and COX-2/PGE2 pathway CGRP expression during OA. ^4^ CGRP is expressed in nerve fibers at both the level of the dorsal root ganglion (DRG) and locally in the knee. ^5^ TLR-4 is often found to be co-localized with TRPV1 leading to rapid neuronal excitation

Figure 4 depicts a molecular pathway that might be involved in gut microbiota-associated OA pain. We understand the complexity of gut microbiome and OA inflammatory and catabolic pathways and thus want to clarify that the pathway is hypothesized based on the published studies conducted in the OA model/patients only. However, considering all the evidence examined up to this point in this review article, targeting gut microbiota through biotic interventions, such as probiotics, represents a novel and potentially fruitful strategy for OA pain management.

## Role of Probiotics in OA Pain: Evidence from Preclinical and Clinical Studies

Probiotics are living organisms detected in food and dietary supplements that provide health benefits to the host when administered in an appropriate dose. Currently, probiotics are live microorganisms that are beneficial to the human body and are of great significance to maintaining the intestinal microecological balance. Probiotics can survive in the passage through the digestive system and can attach to the intestinal epithelia and colonize the gut. Beneficial effects of probiotics are mediated by different mechanisms, including competition against pathogenic bacteria in their binding to the intestinal epithelial cells, enhancement of intestinal epithelial barrier function, inhibition of the growth of pathogens by secreting antimicrobial peptides, and/or enhancement of serum immunoglobulin (Ig) A production, among others [78]. Previous reports have highlighted the role of probiotics in highly prevalent age-related musculoskeletal disorders, likely through restoring gastrointestinal tract barrier functionality and inhibiting pro-inflammatory factors [79]. In addition, several clinical studies have shown that probiotics and their metabolites or probiotic fermented foods have received great attention in improving inflammatory arthritis. Very recently, Zeng et al. performed a systematic review and meta-analysis of 34 randomized controlled trials evaluating the safety and efficacy of probiotic supplementation in 8 types of inflammatory arthritis (hyperuricemia and gout, inflammatory bowel disease arthritis, juvenile idiopathic arthritis, osteoporosis and osteopenia, psoriasis, rheumatoid arthritis, spondyloarthritis, OA) [80••]. The study reported that probiotic supplements might improve these inflammatory arthritides and suggested a need for more randomized controlled trials to determine probiotics’ safety, efficacy, and optimal dosing design.

There is emerging evidence, mostly from preclinical studies, that the administration of probiotics attenuates stress-induced visceral hyperalgesia [81, 82], neuropathic cutaneous mechanical allodynia, and thermal hyperalgesia [83, 84]. Interestingly, clinical studies have also demonstrated the efficacy of probiotics in different pain conditions, such as dental pain [85–87], low back pain [88], menstrual pain [89, 90] and headache/migraine [91–93]. Recent preclinical and clinical studies (Tables 1 and 2) suggest that probiotics may be beneficial to the development of multidisciplinary and multimodal management strategies for treating OA pain.

## Preclinical Studies

The *Lactobacillus casei* group is among some of the most studied species due to their commercial, industrial, and applied health potential [94]. *Lactobacillus casei* has been shown to alleviate inflammatory joint damage in collagen-induced arthritic rats [95]. Oral administration of *Lactobacillus casei* has been proposed as a potent nutraceutical modulator. So et al. found that *Lactobacillus casei*, in combination with type-II collagen and glucosamine, more effectively reduced pain, cartilage destruction, and lymphocyte infiltration than the treatment of glucosamine or *Lactobacillus casei* alone animal model of OA [15]. This co-administration also decreased the expression of the COX-2 enzyme, various pro-inflammatory cytokines (TNF-α, IL-1β, IL-2, IL-6, IL-12, IL-17, and IFN-γ), and matrix metalloproteinases (MMP1, MMP3, and MMP13), while upregulating anti-inflammatory cytokines (IL-4 and IL-10) in both synovial fibroblasts and chondrocytes. The COX-2/PGE2 pathway regulates CGRP expression in the synovial fibroblasts and increasing synovial CGRP levels may contribute to OA pain [96]. Also, the accumulation of MMPs in osteoarthritic cartilage may have a role in OA progression and the biphasic progression of OA-related pain [97]. *Lactobacillus rhamnosus* is known to produce butyrate, a short-chain fatty acid (SCFA), through alteration of the gut microbiota. Oral administration of *Lactobacillus rhamnosus* ameliorated the progression of OA by inhibiting joint pain and inflammation in a rat model of OA [14]. *Lactobacillus rhamnosus* (LR-2)–administered group showed higher levels of expression of peroxisome proliferator-activated receptor gamma (PPAR-γ) and γ-aminobutyric acid (GABA), which are known to control pain, while lower levels of MCP-1 and its receptor, CCR2, which is expected to intensify nervous pain. PPAR-γ ligands have been shown to inhibit major inflammatory signaling pathways in arthritis patients and suppress the COX-2/PGE2 pathway, MMP-1, and IL-6 expression in human synovial fibroblasts [98, 99]. Cingulate GABA levels are inversely correlated with the intensity of ongoing chronic knee OA pain [100]. *Lactobacillus acidophilus* is widely recognized to have probiotic effects and is frequently added to yogurt and fermented milk products. Lee et al. found that inactivated *Lactobacillus acidophilus* (LA-1) can alleviate OA-associated pain and delay the progression of the disease by inhibiting pro-inflammatory cytokine production and reducing cartilage damage [12]. The study also reported that the TRPV1 and CGRP levels in DRG were increased in OA rats but were decreased by *Lactobacillus acidophilus* treatment. Expression of both TRPV1 and CGRP increases during OA, leading to the manifestation of pain [101]. CGRP was expressed in nerve fibers supplying the rat knee at both the level of the DRG and locally in the knee [102]. Cho et al. reported that live LA-1 administration, once daily for 3 weeks, into rats with MIA-induced OA, significantly decreased joint pain by lowering CGRP and TRPV1 expression in DRG and pro-inflammatory factors in the synovial membrane [13]. Interestingly, LA-1 also ameliorated OA-associated intestinal tissue inflammation and altered the gut microbiome. An increase in the level of *Faecalibacterium*, a short-chain fatty acid (SCFA) butyrate-producing bacteria, was reported. Short-chain fatty acids (SCFAs) are the main metabolites produced by the gut microbiota that mediates anti-inflammatory activity by inhibiting histone deacetylation (HDAC) enzyme activity [103, 104]. HDAC enzyme is a crucial regulator of NF-κB activity and pro-inflammatory innate immune response [104]. O-Sullivan et al. studied the effect of live *Lactobacillus acidophilus* (twice weekly; p.o.) in mice with partial medial meniscectomy–induced OA [20]. *Lactobacillus acidophilus* was started 1 week post-surgery to mimic inflammatory pain/early stage of OA. LA treatments showed a significant reduction of pain, TRPV1 expression (in DRG), and pro-inflammatory markers such as TNF-α and NF-κB (in the knee). The study indicated that *Lactobacillus acidophilus* reduced inflammatory knee joint pain and prevented further OA progression when treatment is started at the time of the inflammatory joint pain stage. Furthermore, *Lactobacillus acidophilus* administration significantly enriched the genus *Akkermansia* and bacteria from the Lachnospiraceae family. Lachnospiraceae species hydrolyze starch and other sugars to produce butyrate and other SCFAs [105]. *Lactobacillus plantarum* is a probiotic strain of lactic acid bacteria naturally appearing in the human gut, which can modulate the immune system. Its immunomodulating properties are observed in decreasing the level of anti-inflammatory cytokines. Lin et al. evaluated the therapeutic effects of a live *Lactobacillus plantarum* strain in the anterior cruciate ligament transection (ACLT)–induced OA rat model [18]. *Lactobacillus plantarum* showed a significant reduction in pain-related behavior after 6 weeks of treatment. ACLT-induced synovial inflammation (reduced TNF-α and IL-1β expression) and cartilage damage were minimal in the *Lactobacillus plantarum* group.

*Clostridium butyricum* is a probiotic butyric acid–producing bacterium. Tyndallized *Clostridium butyricum* significantly relieved OA pain, downregulated inflammatory and bone metabolism markers (i.e., COX-2, IL-6, LTB4, and cartilage oligomeric matrix protein), and increased the concentration of IFN-γ [16]. It also acts on matrix metalloproteinases and tissue inhibitors of metalloproteinases (i.e., MMP-2, MMP-3, MMP-9, MMP-13, TIMP-1, and TIMP-2) by inhibiting their mRNA expression. Similarly, *Clostridium butyricum* (GKB7) also improved pain-related behavior from week 2, postoperatively, in anterior cruciate ligament transection (ACLT)–induced OA rats [17]. Moreover, less osseous and cartilaginous damage was observed in week 6. The inflammatory markers IL-1β and TNF-α in cartilage and synovium sections were also reduced significantly. The anti-inflammatory effect of *Clostridium butyricum* mediated via its butyric acid–producing action might be the reason behind its beneficial effects in both animal models of OA. Observing SCFA levels in both models post-probiotic treatment would have been interesting.

*Streptococcus thermophilus* (TCI633) is a newly founded bacterium from human breast milk, and it can produce hyaluronic acid (HA) in the gastrointestinal tract [19]. TCI633 significantly suppressed pain behavior, reduced joint swelling and synovial tissue inflammation, and increased type II collagen expression in the cartilage of an OA rat model. The TCI633 (5 × 10^10^ or 5 × 10^11^ CFU/kg/day) and glucosamine treatment groups showed similar pain relief effects. Previous studies have shown that hyaluronic acid injections can effectively reduce arthritis pain and has therapeutic effects on OA [106, 107].

## Clinical Studies

A small number of human clinical trials have also assessed the therapeutic efficacy of administering probiotics in patients with OA. *Lactobacillus casei* strain Shirota is a well-known probiotic strain that provides health benefits by balancing the gut microbiota and modulating inflammatory and immune responses [108]. A randomized, double-blind clinical trial proposed that the commercial probiotic *Lactobacillus casei* Shirota exhibited beneficial effects on the treatment outcomes of knee joints afflicted with OA. In this study of 537 patients with knee OA, daily supplementation with probiotic *Lactobacillus casei* Shirota over 6 months significantly improved functional scale western Ontario and McMaster universities arthritis index (WOMAC) and pain visual analog scale (VAS) and lowered systemic inflammation (hs-CRP level) compared to placebo [9]. Strong linear correlations were also observed between serum hs-CRP levels, WOMAC, and VAS scores. Elevation of CRP level in OA patients is significantly associated with pain and loss of physical functions [109]. *Lactobacillus casei* Shirota consumption improved pain associated with knee OA, possibly by reducing serum levels of hs-CRP.

In another recent investigation, Lyu et al. conducted a 12-week clinical trial in 80 subjects to validate the efficacy of *Streptococcus thermophilus* (TCI633) in improving the progression of Knee OA [10••]. TCI633 could improve serum collagen type II C-telopeptide (sCTX-II) and serum CRP by 41.58% and 39.58%, respectively, after 12 weeks of treatment. However, the values of pain, stiffness, and function at 0, 4, 8, and 12 weeks in the TCI633 group displayed similar levels with no statistical difference between them. Furthermore, the study reported that the no change in WOMAC scores indicated TCI633-mediated retardation of the OA progression and development at the end of the trial. The authors also highlighted that the indistinct progress of sCTX-II and sCRP might be caused by the uneventful distribution of K/L populations between the TCI633 and placebo groups, a short study period, and few recruited subjects.

Taye et al. performed an N-of-1 trial, divided into three blocks of 10 weeks, to study the efficacy of two daily capsules of *Lactobacillus rhamnosus*, *Saccharomyces cerevisiae*, and *Bifidobacterium animalis* ssp. *lactis* combination in a 67-year-old female with OA in her lower back and right ankle [11••]. The probiotic intervention was associated with lower pain scores and was the preferred intervention chosen by the participant. The mean pain score on the VAS was 4.9 ± 2.2 in the placebo condition compared to 4.0 ± 1.7 in the probiotic condition. The reduction in pain scores associated with the probiotic intervention was small but clinically significant for this patient. This study provides a methodology that individual practitioners can use in clinical practice to generate practice-based evidence.

**Fig. 5:**
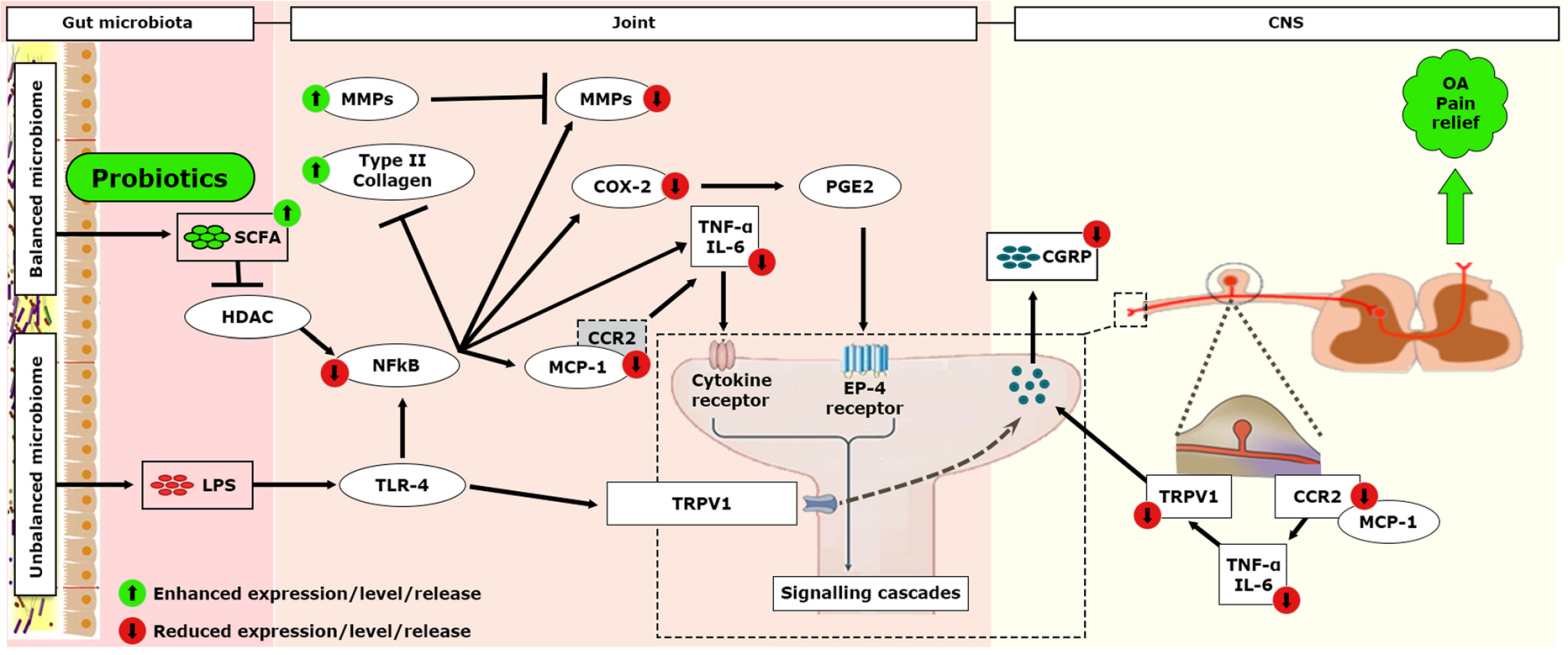
Effect of probiotics in modulating OA-associated pain via multiple signaling pathways connecting the gut, the joint, and the brain

Therefore, based on the published evidence, it can be speculated that probiotics can reduce OA-associated pain via the following mechanisms (Fig. 5).

- Reducing levels of MCP-1, CCR2, TRPV1, and CGRP expression in the dorsal root ganglion (DRG)
- Downregulating MMP expression, COX-2, MCP-1, CCR2, and pro-inflammatory cytokines expression in joint tissues
- Enhancing SCFA level or type II collagen and TIMP-1 expression

However, more high-quality studies are needed to confirm the involvement of these pathways.

## Patent Review and Summary of Ongoing Clinical Trials

Since 2010, 53 patent families disclosed arthritis or OA as an indication for probiotics worldwide (Supplementary Table S1 and Fig. S1). Two innovators hold granted US patents on four probiotic strains, i.e., *Streptococcus thermophilus* (TCI633; US10149868B2), *Lactobacillus acidophilus* CL-92 strain (FERM BP-4981; US9504720B2), *Lactobacillus amylovorus* CP1563 strain (FERM BP-11255; US9504720B2), *Lactobacillus gasseri* CP2305 strain (FERM BP-11331; US9504720B2) for joint pain.

Few registered clinical trials were found that either evaluate the effect of probiotics on OA pain or have yet to post their results. A group of Italian scientists is studying the beneficial effect of probiotic *Lactobacillus casei* in 50 and 40 OA patients for 3 weeks and 3 months, respectively (NCT03968770; NCT03985709). The study focuses on reducing pain intensity along with the changes in the concentrations of pro and anti-inflammatory molecules, including IL-6, TNF-α, soluble IL-6 receptor, soluble type II IL-1 receptor, and CRP. In addition, changes in microbiota composition will be identified through fecal samples using total genomic DNA extraction. The status of the trials has not been provided on clinicaltrial.gov, but they are expected to be completed. Another trial studying the effect of *Lactobacillus rhamnosus* (LGG®; 10 × 10^9^ CFU), *Saccharomyces cerevisiae* (boulardii; 7.5 × 10^9^ CFU), and *Bifidobacterium animalis* ssp. *lactis* (BB-12®; 5 × 10^9^ CFU) combination in OA patients was found to be registered in Australian New Zealand clinical trials registry (ACTRN12621000039886). The primary outcome of the study is to measure the difference in mean pain scores between the placebo and probiotic groups using a numerical rating scale (NRS). As a part of the secondary outcomes, the study will also focus on the difference in the mean score of the functional scale, negative emotional symptoms, personal wellbeing, inflammatory markers (CRP and fibrinogen), and gastrointestinal markers, including zonulin. The trial is completed, and results are expected to be published soon. A trial registered in the Iranian registry of clinical trials is studying the effect of probiotic *Saccharomyces boulardi* on anthropometric indices, functional status, pain and quality of life, and serum indices of inflammation and oxidative stress in overweight and obese patients with knee OA (IRCT20161022030424N4). The trial is undergoing and has not provided information on the expected end date.

## Limitations and Future Perspectives

Despite encouraging results, this review highlights the need for more high-quality clinical studies addressing the potential role of probiotics in OA-related pain. Preclinical animal models only partially mimic the complexity of the human microbiome. Only three clinical studies discussed the role of probiotics in OA. Moreover, most of these studies are short-term (< 6 months). Also, there is no data regarding changes in the microbiome or inflammatory markers for the post-probiotic washout phase. Given the potentially beneficial effect after probiotic supplementation, larger future studies are needed that could further explore gut microbiome diversity by next-generation sequencing. The role of confounding factors also needs to be better explored, in particular genetic background, sex, age, and socio-economic context, including levels of physical activity, diet composition, and use of concomitant prescribed medications.

It is important to note that the mechanisms explored are speculative, as a lack of studies conclusively links pain responses to microbiota and their metabolite-mediated alterations during OA. Therefore, advanced metagenomic and metabolomic-based approaches are imperative to gain mechanistic insight into how gut microbiota and other cellular components interact to produce pain and to understand the therapeutic potential of manipulating the microbiome for pain relief and disease. Our finding concurs with a recent review discussing the need for metatranscriptomic analysis and metabolomic approaches to deeply understand the complexity of the interaction between host and gut microbiome genomes that will help to determine the factors that lead to disease pathogenesis [110•].

Moreover, patients with OA suffer from chronic pain. Thus, it is important to consider bacterial genomic plasticity that determines the influence of different host conditions, such as antibiotic consumption, nutritional influence, and heavy metals from food or drinking water, on probiotic efficacy. This further directed to change in bacterial composition and, thus, the inflammatory response.

One of the main obstacles in chronic pain treatment is the complex mix of pathophysiological and biochemical factors, plus the social, psychological, and economic challenges that patients suffer with their diagnosis. Therefore, assessing the quality of life is a crucial first step in evaluating wellbeing, disease progression, and intervention efficacy. In the case of OA pain, probiotic treatment should focus on more than just reducing pain intensity. It is also essential to consider the effects on the patient’s functional capacity and quality of life [111]. OARSI, the American College of Rheumatology (ACR), the European League of Associations of Rheumatology (EULAR), and the Initiative on Methods, Measurement, and Pain Assessment in Clinical Trials (IMMPACT) have each issued recommendations or reports indicating how researchers should conduct OA-related studies. While these reports typically used outcome measures that examine pain, functional ability, and global patient assessments, these publications also noted the need for better, validated outcome measures to examine the quality of life [112–115].

Currently, sequencing technologies, usually based on samples collected from feces, mucosal biopsy, intestinal fluid, etc., have enabled researchers to explore and understand the gut microbiome from a broader and deeper perspective. However, the technique has its limitations, such as fecal samples being just a proxy for intestinal microbiota, while biopsies are invasive for patients and unsuitable for healthy controls. Given the availability of large‐scale biobanks such as the UK Biobank and worldwide collaborative efforts, genome-wide association studies (GWAS) power has increased in recent years. Yu et al. utilized large-scale GWAS summary statistics for OA generated by meta-analysis of the UK Biobank and arcOGEN to evaluate the causal association between gut microbiota and OA risk [116]. They found a protective role of multiple gut microbiota in the development of OA. Such studies give researchers a direction that can be explored to improve understanding of a complex microbiome and OA. However, many challenges remain in establishing causal relationships using current genetic data and approaches. Firstly, the multivariable Mendelian randomization analysis ruled out the pleiotropic effects of BMI that exist between causal gut microbiota and the development of OA. The statistically insignificant results for suggestive gut microbiota could be attributed to limited statistical power or potential BMI-related pleiotropic effects. Secondly, there is a lack of reliable single-nucleotide polymorphisms (SNPs) associated with the gut microbiome. Thirdly, the biological function through which the SNPs influence the gut microbiome is unsure and complex, especially considering the possible bilateral nature of the association within the GWAS exposure (i.e., the human microbiome influencing health status and health status influencing the human microbiome). Finally, due to the lack of individual data, it is not possible to conduct further population stratification studies (e.g., gender) and explore possible differences in different populations.

## Conclusions

The connection between gut microbiota imbalance and its role in the initiation and progression of OA and associated pain has been increasingly recognized. Experimental data from preclinical studies and emerging trends from clinical studies strongly suggest that probiotics may benefit patients with OA pain through positive gut microbiota modulation and attenuating low-grade inflammation via multiple pathways. Patent data also suggests increased interest in the development of novel nutritional innovations that involve probiotic use for reducing OA and joint pain. The strain selection is the most important factor for determining positive impact, specifically on improving the OA symptoms. Further advancement of knowledge in this area will undoubtedly pave the way for the development of probiotic strains that can be used to improve treatment outcomes in OA and reduce the huge impact of this serious disease on healthcare systems worldwide. The influx of this novel concept along with cutting-edge research added a new perspective on the management of OA which further adds a novel direction in the development of multidisciplinary and multimodal management strategies for treating OA pain.

### Supplementary Information

Below is the link to the electronic supplementary material.

Supplementary file1 (DOCX 148 KB)

## Data Availability

The data from this study will be made available upon request after publication of the study.

## References

[1] GBD 2017 Disease and Injury Incidence and Prevalence Collaborators (2018). Global, regional, and national incidence, prevalence, and years lived with disability for 354 diseases and injuries for 195 countries and territories, 1990–2017: a systematic analysis for the Global Burden of Disease Study 2017. Lancet (London, England)..

[2] Abramoff B, Caldera FE (2020). Osteoarthritis: pathology, diagnosis, and treatment options. Med Clin North Am.

[3] Terkawi MA, Ebata T, Yokota S, Takahashi D, Endo T, Matsumae G, et al. Low-grade inflammation in the pathogenesis of osteoarthritis: cellular and molecular mechanisms and strategies for future therapeutic intervention. Biomedicines. 2022;10(5). 10.3390/biomedicines10051109. 10.3390/biomedicines10051109 PMC9139060 35625846

[4] Woodell-May JE, Sommerfeld SD (2020). Role of Inflammation and the immune system in the progression of osteoarthritis. J Orthopaedic Res : Off Publ Orthopaedic Res Soc.

[5] Miller RJ, Malfait AM, Miller RE (2020). The innate immune response as a mediator of osteoarthritis pain. Osteoarthritis Cartilage.

[6] Durack J, Lynch SV (2019). The gut microbiome: relationships with disease and opportunities for therapy. J Exp Med.

[7] Hao X, Shang X, Liu J, Chi R, Zhang J, Xu T (2021). The gut microbiota in osteoarthritis: where do we stand and what can we do?. Arthritis Res Ther.

[8.••] Guo R, Chen LH, Xing C, Liu T (2019). Pain regulation by gut microbiota: molecular mechanisms and therapeutic potential. British journal of anaesthesia..

[9] Lei M, Guo C, Wang D, Zhang C, Hua L (2017). The effect of probiotic Lactobacillus casei Shirota on knee osteoarthritis: a randomised double-blind, placebo-controlled clinical trial. Beneficial Microbes..

[10.••] Lyu JL, Wang TM, Chen YH, Chang ST, Wu MS, Lin YH (2020). Oral intake of Streptococcus thermophil us improves knee osteoarthritis degeneration: a randomized, double-blind, placebo-controlled clinical study. Heliyon..

[11.••] Taye I, Bradbury J, Grace S, Avila C (2020). Probiotics for pain of osteoarthritis; an N-of-1 trial of individual effects. Complement Ther Med.

[12] Lee SH, Kwon JY, Jhun J, Jung K, Park SH, Yang CW (2018). Lactobacillus acidophilus ameliorates pain and cartilage degradation in experimental osteoarthritis. Immunol Lett.

[13] Cho KH, Na HS, Jhun J, Woo JS, Lee AR, Lee SY (2022). Lactobacillus (LA-1) and butyrate inhibit osteoarthritis by controlling autophagy and inflammatory cell death of chondrocytes. Front Immunol.

[14] Jhun J, Cho KH, Lee DH, Kwon JY, Woo JS, Kim J, et al. Oral administration of Lactobacillus rhamnosus ameliorates the progression of osteoarthritis by inhibiting joint pain and inflammation. Cells. 2021;10(5). 10.3390/cells10051057. 10.3390/cells10051057 PMC8146916 33946919

[15] So JS, Song MK, Kwon HK, Lee CG, Chae CS, Sahoo A (2011). Lactobacillus casei enhances type II collagen/glucosamine-mediated suppression of inflammatory responses in experimental osteoarthritis. Life Sci.

[16] Sim BY, Choi HJ, Kim MG, Jeong DG, Lee DG, Yoon JM (2018). Effects of ID-CBT5101 in preventing and alleviating osteoarthritis symptoms in a monosodium iodoacetate-induced rat model. J Microbiol Biotechnol.

[17] Chang SL, Lin YY, Liu SC, Tsai YS, Lin SW, Chen YL, et al. Oral administration of Clostridium butyricum GKB7 ameliorates signs of osteoarthritis in rats. Cells. 2022;11(14). 10.3390/cells11142169 10.3390/cells11142169 PMC9323988 35883610

[18] Lin YY, Chang SL, Liu SC, Achudhan D, Tsai YS, Lin SW, et al. Therapeutic effects of live Lactobacillus plantarum GKD7 in a rat model of knee osteoarthritis. Nutrients. 2022;14(15). 10.3390/nu14153170 10.3390/nu14153170 PMC9370768 35956346

[19] Lin YY, Chen NF, Yang SN, Jean YH, Kuo HM, Chen PC (2021). Effects of Streptococcus thermophilus on anterior cruciate ligament transection-induced early osteoarthritis in rats. Exp Ther Med.

[20] I OS, Natarajan Anbazhagan A, Singh G, Ma K, Green SJ, Singhal M, et al. Lactobacillus acidophilus mitigates osteoarthritis-associated pain, cartilage disintegration and gut microbiota dysbiosis in an experimental murine OA model. Biomedicines. 2022;10(6). 10.3390/biomedicines10061298. 10.3390/biomedicines10061298 PMC9220766 35740320

[21] Benito MJ, Veale DJ, FitzGerald O, van den Berg WB, Bresnihan B (2005). Synovial tissue inflammation in early and late osteoarthritis. Ann Rheum Dis.

[22] Sokolove J, Lepus CM (2013). Role of inflammation in the pathogenesis of osteoarthritis: latest findings and interpretations. Ther Adv Musculoskeletal Dis.

[23] Pauli C, Grogan SP, Patil S, Otsuki S, Hasegawa A, Koziol J (2011). Macroscopic and histopathologic analysis of human knee menisci in aging and osteoarthritis. Osteoarthritis Cartilage.

[24] Robinson WH, Lepus CM, Wang Q, Raghu H, Mao R, Lindstrom TM (2016). Low-grade inflammation as a key mediator of the pathogenesis of osteoarthritis. Nat Rev Rheumatol.

[25] Lieberthal J, Sambamurthy N, Scanzello CR (2015). Inflammation in joint injury and post-traumatic osteoarthritis. Osteoarthritis Cartilage.

[26] Gómez R, Villalvilla A, Largo R, Gualillo O, Herrero-Beaumont G (2015). TLR4 signalling in osteoarthritis–finding targets for candidate DMOADs. Nat Rev Rheumatol.

[27] Roh JS, Sohn DH (2018). Damage-associated molecular patterns in inflammatory diseases. Immune network..

[28] Kawai T, Akira S (2007). Signaling to NF-kappaB by Toll-like receptors. Trends Mol Med.

[29] Suantawee T, Tantavisut S, Adisakwattana S, Tanpowpong T, Tanavalee A, Yuktanandana P (2015). Upregulation of inducible nitric oxide synthase and nitrotyrosine expression in primary knee osteoarthritis. J Med Assoc Thailand Chotmaihet thangphaet..

[30] Liu YX, Wang GD, Wang X, Zhang YL, Zhang TL (2017). Effects of TLR-2/NF-κB signaling pathway on the occurrence of degenerative knee osteoarthritis an in vivo and in vitro study. Oncotarget..

[31] Ostojic M, Zevrnja A, Vukojevic K, Soljic V. Immunofluorescence analysis of NF-kB and iNOS expression in different cell populations during early and advanced knee osteoarthritis. Int J Mole Sci. 2021;22(12). 10.3390/ijms22126461. 10.3390/ijms22126461 PMC8233870 34208719

[32] Nedunchezhiyan U, Varughese I, Sun AR, Wu X, Crawford R, Prasadam I (2022). Obesity, inflammation, and immune system in osteoarthritis. Frontiers in immunology..

[33] Smith JW, Martins TB, Gopez E, Johnson T, Hill HR, Rosenberg TD (2012). Significance of C-reactive protein in osteoarthritis and total knee arthroplasty outcomes. Ther Adv Musculoskeletal Dis.

[34] Snelling SJ, Bas S, Puskas GJ, Dakin SG, Suva D, Finckh A (2017). Presence of IL-17 in synovial fluid identifies a potential inflammatory osteoarthritic phenotype. PloS one..

[35] Pearle AD, Scanzello CR, George S, Mandl LA, DiCarlo EF, Peterson M (2007). Elevated high-sensitivity C-reactive protein levels are associated with local inflammatory findings in patients with osteoarthritis. Osteoarthritis Cartilage.

[36.•] Chisari E, Wouthuyzen-Bakker M, Friedrich AW, Parvizi J (2021). The relation between the gut microbiome and osteoarthritis: a systematic review of literature. PloS one..

[37] Tilg H, Zmora N, Adolph TE, Elinav E (2020). The intestinal microbiota fuelling metabolic inflammation. Nat Rev Immunol.

[38] Manichanh C, Borruel N, Casellas F, Guarner F (2012). The gut microbiota in IBD. Nat Rev Gastroenterol Hepatol.

[39] de Kort S, Keszthelyi D, Masclee AA (2011). Leaky gut and diabetes mellitus: what is the link?. Obesity Rev : Off J Int Assoc Study Obesity.

[40] Anto L, Blesso CN (2022). Interplay between diet, the gut microbiome, and atherosclerosis: role of dysbiosis and microbial metabolites on inflammation and disordered lipid metabolism. J Nutri Biochem..

[41.•] Tonelli Enrico V, Vo N, Methe B, Morris A, Sowa G (2022). An unexpected connection: a narrative review of the associations between gut microbiome and musculoskeletal pain. Eur Spine J : Off Publ Eur Spine Soc, Eur Spinal Deform Soc, Eur Sect Cervical Spine Res Soc.

[42] Guo S, Al-Sadi R, Said HM, Ma TY (2013). Lipopolysaccharide causes an increase in intestinal tight junction permeability in vitro and in vivo by inducing enterocyte membrane expression and localization of TLR-4 and CD14. Am J Pathol.

[43] Huang Z, Kraus VB (2016). Does lipopolysaccharide-mediated inflammation have a role in OA?. Nat Rev Rheumatol.

[44] Tanaka S, Toki T, Akimoto T, Morishita K (2013). Lipopolysaccharide accelerates collagen-induced arthritis in association with rapid and continuous production of inflammatory mediators and anti-type II collagen antibody. Microbiol Immunol.

[45] Islam MR, Arthur S, Haynes J, Butts MR, Nepal N, Sundaram U. The role of gut microbiota and metabolites in obesity-associated chronic gastrointestinal disorders. Nutrients. 2022;14(3). 10.3390/nu14030624. 10.3390/nu14030624 PMC8838694 35276983

[46] Rohr MW, Narasimhulu CA, Rudeski-Rohr TA, Parthasarathy S (2020). Negative effects of a high-fat diet on intestinal permeability: a review. Adv Nutri (Bethesda, Md).

[47] Metcalfe D, Harte AL, Aletrari MO, Al Daghri NM, Al Disi D, Tripathi G (2012). Does endotoxaemia contribute to osteoarthritis in obese patients?. Clin Sci (London England: 1979).

[48] Chen Y, Jiang W, Yong H, He M, Yang Y, Deng Z (2020). Macrophages in osteoarthritis: pathophysiology and therapeutics. Am J Trans Res.

[49] Bode JG, Ehlting C, Häussinger D (2012). The macrophage response towards LPS and its control through the p38(MAPK)-STAT3 axis. Cell Signal.

[50] Mendez ME, Murugesh DK, Sebastian A, Hum NR, McCloy SA, Kuhn EA, et al. Antibiotic treatment prior to injury improves post-traumatic osteoarthritis outcomes in mice. International journal of molecular sciences. 2020;21(17). 10.3390/ijms21176424. 10.3390/ijms21176424 PMC7503363 32899361

[51.••] Guan Z, Jia J, Zhang C, Sun T, Zhang W, Yuan W (2020). Gut microbiome dysbiosis alleviates the progression of osteoarthritis in mice. Clinical science (London, England : 1979).

[52] Yu LP, Smith GN, Brandt KD, Myers SL, O’Connor BL, Brandt DA (1992). Reduction of the severity of canine osteoarthritis by prophylactic treatment with oral doxycycline. Arthritis Rheum.

[53] Brandt KD, Mazzuca SA, Katz BP, Lane KA, Buckwalter KA, Yocum DE (2005). Effects of doxycycline on progression of osteoarthritis: results of a randomized, placebo-controlled, double-blind trial. Arthritis Rheum.

[54] Hameed S (2019). Na(v)1.7 and Na(v)1.8: Role in the pathophysiology of pain. Mole Pain.

[55] Miller RE, Tran PB, Das R, Ghoreishi-Haack N, Ren D, Miller RJ (2012). CCR2 chemokine receptor signaling mediates pain in experimental osteoarthritis. Proc Natl Acad Sci USA.

[56] Fingleton C, Smart K, Moloney N, Fullen BM, Doody C (2015). Pain sensitization in people with knee osteoarthritis: a systematic review and meta-analysis. Osteoarthritis Cartilage.

[57] Höper J, Schraml L, Gierthmühlen J, Helfert SM, Rehm S, Härtig S, et al. Changes of somatosensory phenotype in the course of disease in osteoarthritis patients. International journal of environmental research and public health. 2020;17(9). 10.3390/ijerph17093085. 10.3390/ijerph17093085 PMC7246567 32365479

[58.••] Morreale C, Bresesti I, Bosi A, Baj A, Giaroni C, Agosti M, et al. Microbiota and pain: save your gut feeling. Cells. 2022;11(6). 10.3390/cells11060971. **A recent and important review highlighting role of gut-brain axis and efficacy of probiotics in different pain disorders.** 10.3390/cells11060971 PMC8946251 35326422

[59] Diogenes A, Ferraz CC, Akopian AN, Henry MA, Hargreaves KM (2011). LPS sensitizes TRPV1 via activation of TLR4 in trigeminal sensory neurons. J Dent Res.

[60] Qi J, Buzas K, Fan H, Cohen JI, Wang K, Mont E (2011). Painful pathways induced by TLR stimulation of dorsal root ganglion neurons. J Immunol (Baltimore, Md : 1950)..

[61] Meseguer V, Alpizar YA, Luis E, Tajada S, Denlinger B, Fajardo O (2014). TRPA1 channels mediate acute neurogenic inflammation and pain produced by bacterial endotoxins. Nat Commun.

[62] Chen G, Zhang YQ, Qadri YJ, Serhan CN, Ji RR (2018). Microglia in pain: detrimental and protective roles in pathogenesis and resolution of pain. Neuron.

[63] Gao YJ, Ji RR (2010). Chemokines, neuronal-glial interactions, and central processing of neuropathic pain. Pharmacol Ther.

[64] Matsuda M, Huh Y, Ji RR (2019). Roles of inflammation, neurogenic inflammation, and neuroinflammation in pain. J Anesth.

[65] Latremoliere A, Woolf CJ (2009). Central sensitization: a generator of pain hypersensitivity by central neural plasticity. J Pain.

[66] Abdel-Haq R, Schlachetzki JCM, Glass CK, Mazmanian SK (2019). Microbiome-microglia connections via the gut-brain axis. J Exp Med.

[67] Erny D, Hrabě de Angelis AL, Jaitin D, Wieghofer P, Staszewski O, David E (2015). Host microbiota constantly control maturation and function of microglia in the CNS. Nat Neurosci.

[68.•] Dahshan D, Gallagher N, Workman A, Perdue J, Aikens J, Schmicker T (2022). Targeting the gut microbiome for inflammation and pain management in orthopedic conditions. Orthopedics.

[69] Amaral FA, Sachs D, Costa VV, Fagundes CT, Cisalpino D, Cunha TM (2008). Commensal microbiota is fundamental for the development of inflammatory pain. Proc Natl Acad Sci USA.

[70] Yan S, Kentner AC (2017). Mechanical allodynia corresponds to Oprm1 downregulation within the descending pain network of male and female rats exposed to neonatal immune challenge. Brain Behav Immun.

[71] Vieira AT, Macia L, Galvão I, Martins FS, Canesso MC, Amaral FA (2015). A role for gut microbiota and the metabolite-sensing receptor GPR43 in a murine model of gout. Arthritis Rheumatol (Hoboken, NJ).

[72.••] Sánchez Romero EA, Meléndez Oliva E, Alonso Pérez JL, Martín Pérez S, Turroni S, Marchese L, et al. Relationship between the gut microbiome and osteoarthritis pain: review of the literature. Nutrients. 2021;13(3). 10.3390/nu13030716. **A systematic review highlighting how dysbiosis and a proinflammatory microbiome profile in OA patients might play a role in the severity of symptoms, particularly pain.** 10.3390/nu13030716 PMC7996179 33668236

[73] Boer CG, Radjabzadeh D, Medina-Gomez C, Garmaeva S, Schiphof D, Arp P (2019). Intestinal microbiome composition and its relation to joint pain and inflammation. Nat Commun.

[74] Wu HJ, Ivanov II, Darce J, Hattori K, Shima T, Umesaki Y (2010). Gut-residing segmented filamentous bacteria drive autoimmune arthritis via T helper 17 cells. Immunity.

[75] Huang ZY, Stabler T, Pei FX, Kraus VB (2016). Both systemic and local lipopolysaccharide (LPS) burden are associated with knee OA severity and inflammation. Osteoarthritis Cartilage.

[76.••] Dunn CM, Velasco C, Rivas A, Andrews M, Garman C, Jacob PB (2020). Identification of cartilage microbial DNA signatures and associations with knee and hip osteoarthritis. Arthritis & rheumatology (Hoboken, NJ).

[77.•] Favazzo LJ, Hendesi H, Villani DA, Soniwala S, Dar QA, Schott EM (2020). The gut microbiome-joint connection: implications in osteoarthritis. Curr Opin Rheumatol.

[78] Wieërs G, Belkhir L, Enaud R, Leclercq S, Philippart de Foy JM, Dequenne I (2019). How probiotics affect the microbiota. Front Cell Infect Microbiol.

[79] Han S, Lu Y, Xie J, Fei Y, Zheng G, Wang Z (2021). Probiotic gastrointestinal transit and colonization after oral administration: a long journey. Front Cell Infect Microbiol.

[80.••] Zeng L, Deng Y, He Q, Yang K, Li J, Xiang W (2022). Safety and efficacy of probiotic supplementation in 8 types of inflammatory arthritis: a systematic review and meta-analysis of 34 randomized controlled trials. Front Immunol.

[81] Ford AC, Quigley EM, Lacy BE, Lembo AJ, Saito YA, Schiller LR, et al. Efficacy of prebiotics, probiotics, and synbiotics in irritable bowel syndrome and chronic idiopathic constipation: systematic review and meta-analysis. Am J Gastroenterol. 2014;109(10):1547–61; quiz 6, 62. 10.1038/ajg.2014.202. 10.1038/ajg.2014.202 25070051

[82] McVey Neufeld KA, Strain CR, Pusceddu MM, Waworuntu RV, Manurung S, Gross G (2020). Lactobacillus rhamnosus GG soluble mediators ameliorate early life stress-induced visceral hypersensitivity and changes in spinal cord gene expression. Neuronal Signal..

[83] Cuozzo M, Castelli V, Avagliano C, Cimini A, d’Angelo M, Cristiano C, et al. Effects of chronic oral probiotic treatment in paclitaxel-induced neuropathic pain. Biomedicines. 2021;9(4). 10.3390/biomedicines9040346. 10.3390/biomedicines9040346 PMC8066538 33808052

[84] Ding W, You Z, Chen Q, Yang L, Doheny J, Zhou X (2021). Gut microbiota influences neuropathic pain through modulating proinflammatory and anti-inflammatory T cells. Anesth Analg.

[85] Banjonjit S, Taweechotipatr M, Rungsiyanont S (2022). Effect of probiotic Lactobacillus paracasei on tumor necrosis factor-alpha level in gingival crevicular fluid of patients undergoing impacted third molar removal. J Oral Sci.

[86] Ferrés-Amat E, Espadaler-Mazo J, Calvo-Guirado JL, Ferrés-Amat E, Mareque-Bueno J, Salavert A (2020). Probiotics diminish the post-operatory pain following mandibular third molar extraction: a randomised double-blind controlled trial (pilot study). Beneficial Microbes.

[87] Wälivaara D, Sjögren I, Gerasimcik N, Yucel-Lindberg T, Twetman S, Abrahamsson P (2019). Effects of Lactobacillus reuteri-containing lozenges on healing after surgical removal of mandibular third molars: a randomised controlled trial. Beneficial Microbes.

[88] Jensen OK, Andersen MH, Østgård RD, Andersen NT, Rolving N (2019). Probiotics for chronic low back pain with type 1 Modic changes: a randomized double-blind, placebo-controlled trial with 1-year follow-up using Lactobacillus Rhamnosis GG. Eur Spine J : Off Publ Eur Spine Soc, Eur Spinal Deform Soc, Eur Sect Cervical Spine Res Soc.

[89] Itoh H, Uchida M, Sashihara T, Ji ZS, Li J, Tang Q (2011). Lactobacillus gasseri OLL2809 is effective especially on the menstrual pain and dysmenorrhea in endometriosis patients: randomized, double-blind, placebo-controlled study. Cytotechnology.

[90] Khodaverdi S, Mohammadbeigi R, Khaledi M, Mesdaghinia L, Sharifzadeh F, Nasiripour S (2019). Beneficial effects of oral lactobacillus on pain severity in women suffering from endometriosis: a pilot placebo-controlled randomized clinical trial. Int J Fertil Steril.

[91] Ghavami A, Khorvash F, Heidari Z, Khalesi S, Askari G (2021). Effect of synbiotic supplementation on migraine characteristics and inflammatory biomarkers in women with migraine: results of a randomized controlled trial. Pharmacol Res..

[92] Martami F, Togha M, Seifishahpar M, Ghorbani Z, Ansari H, Karimi T (2019). The effects of a multispecies probiotic supplement on inflammatory markers and episodic and chronic migraine characteristics: a randomized double-blind controlled trial. Cephalalgia : Int J Headache.

[93] Qi X, Fan G, Jia H (2020). The probiotic Lactobacillus casei Shirota attenuates symptoms of vestibular migraine: a randomised placebo-controlled double-blind clinical trial. Beneficial Microbes.

[94] Hill D, Sugrue I, Tobin C, Hill C, Stanton C, Ross RP (2018). The Lactobacillus casei group: history and health related applications. Front Microbiol.

[95] Amdekar S, Singh V, Singh R, Sharma P, Keshav P, Kumar A (2011). Lactobacillus casei reduces the inflammatory joint damage associated with collagen-induced arthritis (CIA) by reducing the pro-inflammatory cytokines: Lactobacillus casei: COX-2 inhibitor. J Clin Immunol.

[96] Takano S, Uchida K, Inoue G, Minatani A, Miyagi M, Aikawa J (2017). Increase and regulation of synovial calcitonin gene-related peptide expression in patients with painful knee osteoarthritis. J Pain Res.

[97] Pajak A, Kostrzewa M, Malek N, Korostynski M, Starowicz K (2017). Expression of matrix metalloproteinases and components of the endocannabinoid system in the knee joint are associated with biphasic pain progression in a rat model of osteoarthritis. J Pain Res.

[98] Farrajota K, Cheng S, Martel-Pelletier J, Afif H, Pelletier JP, Li X (2005). Inhibition of interleukin-1beta-induced cyclooxygenase 2 expression in human synovial fibroblasts by 15-deoxy-Delta 12,14-prostaglandin J2 through a histone deacetylase-independent mechanism. Arthritis Rheum.

[99] Giaginis C, Giagini A, Theocharis S (2009). Peroxisome proliferator-activated receptor-gamma (PPAR-gamma) ligands as potential therapeutic agents to treat arthritis. Pharmacol Res.

[100] Reckziegel D, Raschke F, Cottam WJ, Auer DP. Cingulate GABA levels inversely correlate with the intensity of ongoing chronic knee osteoarthritis pain. Mole Pain. 2016;12. 10.1177/1744806916650690. 10.1177/1744806916650690 PMC4956171 27206661

[101] Fernihough J, Gentry C, Bevan S, Winter J (2005). Regulation of calcitonin gene-related peptide and TRPV1 in a rat model of osteoarthritis. Neurosci Lett.

[102] Grässel SG (2014). The role of peripheral nerve fibers and their neurotransmitters in cartilage and bone physiology and pathophysiology. Arthritis Res Ther.

[103] Markowiak-Kopeć P, Śliżewska K. The effect of probiotics on the production of short-chain fatty acids by human intestinal microbiome. Nutrients. 2020;12(4). 10.3390/nu12041107. 10.3390/nu12041107 PMC7230973 32316181

[104] Rooks MG, Garrett WS (2016). Gut microbiota, metabolites and host immunity. Nat Rev Immunol.

[105] Vacca M, Celano G, Calabrese FM, Portincasa P, Gobbetti M, De Angelis M. The controversial role of human gut Lachnospiraceae. Microorganisms. 2020;8(4). 10.3390/microorganisms8040573 10.3390/microorganisms8040573 PMC7232163 32326636

[106] Hashizume M, Koike N, Yoshida H, Suzuki M, Mihara M (2010). High molecular weight hyaluronic acid relieved joint pain and prevented the progression of cartilage degeneration in a rabbit osteoarthritis model after onset of arthritis. Mod Rheumatol.

[107] Monticone M, Frizziero A, Rovere G, Vittadini F, Uliano D, Lab S (2016). Hyaluronic acid intra-articular injection and exercise therapy: effects on pain and disability in subjects affected by lower limb joints osteoarthritis A systematic review by the Italian Society of Physical and Rehabilitation Medicine (SIMFER). Eur J Phys Rehabilitat Med.

[108] Kato-Kataoka A, Nishida K, Takada M, Kawai M, Kikuchi-Hayakawa H, Suda K (2016). Fermented milk containing Lactobacillus casei strain Shirota preserves the diversity of the gut microbiota and relieves abdominal dysfunction in healthy medical students exposed to academic stress. Appl Environ Microbiol.

[109] Jin X, Beguerie JR, Zhang W, Blizzard L, Otahal P, Jones G (2015). Circulating C reactive protein in osteoarthritis: a systematic review and meta-analysis. Ann Rheum Dis.

[110.•] Tan TC, Chong TKY, Low AHL, Leung YY (2021). Microbiome and osteoarthritis: new insights from animal and human studies. Int J Rheum Dis.

[111] Bagraith KS, Strong J, Meredith PJ, McPhail SM (2018). What do clinicians consider when assessing chronic low back pain? A content analysis of multidisciplinary pain centre team assessments of functioning, disability, and health. Pain.

[112] Altman R, Brandt K, Hochberg M, Moskowitz R, Bellamy N, Bloch DA (1996). Design and conduct of clinical trials in patients with osteoarthritis: recommendations from a task force of the Osteoarthritis Research Society Results from a workshop. Osteoarthritis Cartilage..

[113] Bellamy N, Kirwan J, Boers M, Brooks P, Strand V, Tugwell P (1997). Recommendations for a core set of outcome measures for future phase III clinical trials in knee, hip, and hand osteoarthritis Consensus development at OMERACT III. J Rheumatol.

[114] Jordan KM, Arden NK, Doherty M, Bannwarth B, Bijlsma JW, Dieppe P (2003). EULAR recommendations 2003: an evidence based approach to the management of knee osteoarthritis: report of a task force of the Standing Committee for International Clinical Studies Including Therapeutic Trials (ESCISIT). Ann Rheum Dis.

[115] Smith TO, Hawker GA, Hunter DJ, March LM, Boers M, Shea BJ (2019). The OMERACT-OARSI core domain set for measurement in clinical trials of hip and/or knee osteoarthritis. J Rheumatol.

[116] Yu XH, Yang YQ, Cao RR, Bo L, Lei SF (2021). The causal role of gut microbiota in development of osteoarthritis. Osteoarthritis Cartilage.

