# Supplementary material for: The Potential Role of Probiotics in the Management of Osteoarthritis Pain: Current Status and Future Prospects
Source: Curr Rheumatol Rep. 2023 Sep 1;25(12):307–26. doi: 10.1007/s11926-023-01108-7 (PMC10754743; doi:10.1007/s11926-023-01108-7)
Supplement: Supplementary file 1 — Supplementary file1 (DOCX 148 KB) [file 11926_2023_1108_MOESM1_ESM.docx]

**Supplementary table**

**Figure S1**: 53 Patent families disclosed arthritis, osteoarthritis or joint pain as an indication for probiotics


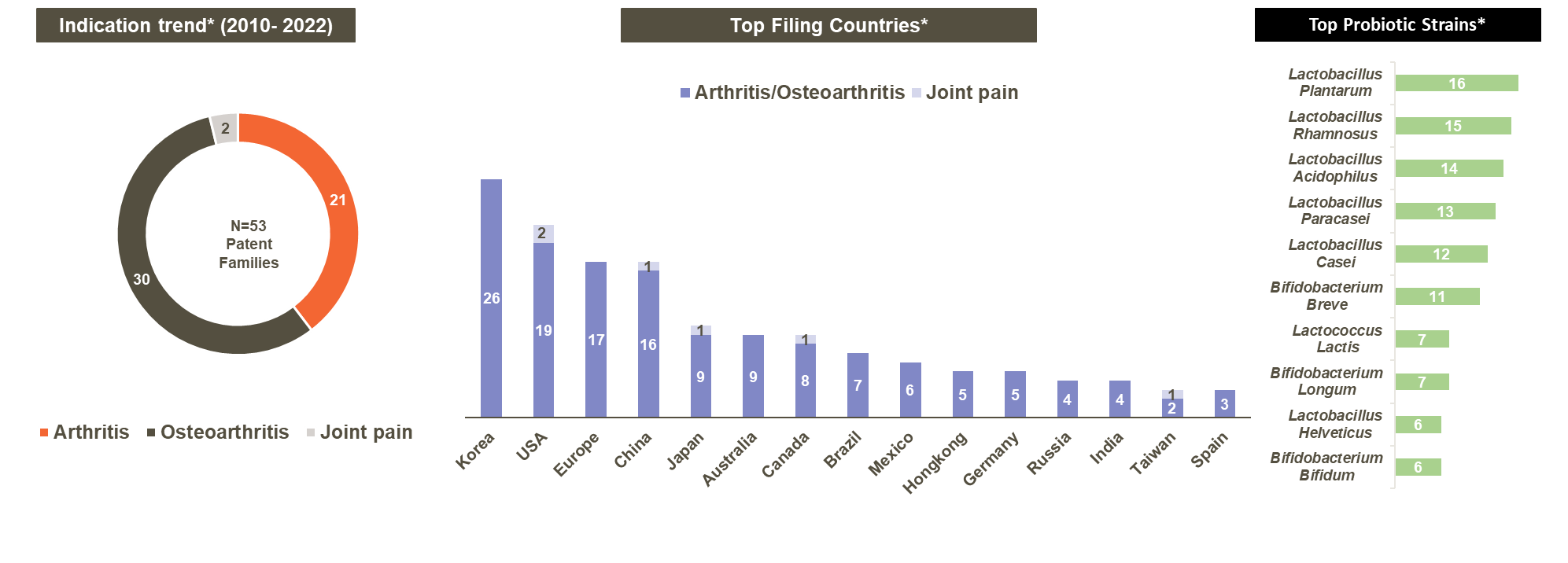


**Table S1**: Patent filled/granted on probiotics for arthritis, osteoarthritis or joint pain identified from Jan 2010 – Mar 2022 (Earliest publication date)

| **Patent Number** | **Company/ Assignee Name** | **Company/ University/ Individual** | **Probiotic strain (s)** | **Product Format (s)** | **Legal Status** | | **Geography** |
| --- | --- | --- | --- | --- | --- | --- | --- |
|  |  |  |  |  | **Standard** | **Details** |  |
| US9504720B2 | Asahi Group Holdings Ltd | Company | *Lactobacillus acidophilus CL-92 strain (FERM BP-4981), Lactobacillus amylovorus CP1563 strain (FERM BP-11255), Lactobacillus gasseri CP2305 strain (FERM BP-11331)* | Drink | Granted | Granted; Expected Expiry - 23rd Apr 2033 | Canada, Japan, USA, PCT* |
| US10149868B2 | Tci Co Ltd | Company | *Streptococcus thermophilus TCI633* | Powder, tablet, dragee, chewable tablet, pill, capsule, granule, pulvis, fluidextract, solution, syrup, suspension, tincture | Granted | Granted; Expected Expiry - 30th May 2037 | China, Taiwan, USA |
| US9468659B2 | Nwo Stem Cure Llc | Company | *Lactobacillus rhamnosus* | Tablets, capsules, liquid | Granted | Granted; Expected Expiry - 6th Nov 2034 | USA |
| KR101941183B1 | Bioport Korea Inc | Company | *Lactobacillus casei, Lactobacillus rhamnosus, Bifidobacterium bifidum, Bifidobacterium breve, Lactobacillus acidophilus, Bacillus subtilis* | Tablet pills, powders, granules, capsules | Granted | Granted; Expected Expiry - 24th Oct 2036 | South Korea |
| KR101770036B1 | Dong Eui Univ Ind Academic Coop Found | Academia | *Lactobacillus casei, Lactobacillus rhamnosus, Bifidobacterium bifidum bifidus, Bifidobacterium breve bifidobacterium, Lactobacillus acidophilus* | Tablet, pills, powders, granules, capsules, suspensions, solutions, emulsions, syrups, sterilized aqueous solutions | Granted | Granted; Expected Expiry - 17th Oct 2035 | South Korea |
| KR101770035B1 | Dong Eui Univ Ind Academic Coop Found | Academia | *Lactobacillus casei, Lactobacillus rhamnosus, Bifidobacterium bifidum bifidus, Bifidobacterium breve bifidobacterium, Lactobacillus acidophilus* | Tablet, pills, powders, granules, capsules, suspensions, solutions, emulsions, syrups, sterilized aqueous solutions | Granted | Granted; Expected Expiry - 17th Oct 2035 | South Korea |
| KR101928881 B1 | Mediandbio Co Ltd | Company | *Lactobacillus plantarum, Leuconostoc, Pediococcus, Lactococcus, Streptococcus, Enterococcus, Bifidobacterium, Bifidobacterium, Sporolactobacillus, Weissella* | Powders, granules, tablets, capsules, gels, solutions, suspensions, emulsions, oils | Granted | Granted; Expected Expiry - 3rd Oct 2037 | South Korea |
| KR101338941B1 | NUC Life & Health Co. | Company | *Bacillus subtilis BN-NUC1* | Capsule | Granted | Granted; Expected Expiry - 25th Jul 2031 | South Korea |
| KR102044659 B1 | Sk Bioland Co Ltd | Company | *Lactobacillus plantarum SKB1234* | Powders, granules, tablets, capsules, suspensions, emulsions, syrups, aerosols | Granted | Granted; Expected Expiry - 26th Apr 2039 | South Korea |
| KR102007144 B1 | Sk Bioland Co Ltd | Company | *Lactobacillus plantarum SKB1234* | Powders, granules, tablets, capsules, suspensions, emulsions, syrups, aerosols | Granted | Granted; Expected Expiry - 26th Apr 2039 | South Korea |
| IN349711 B | Stelis Biopharma | Company | *Streptococcus equi subsp. zooepidemicus ATCC 35246* | Solid, liquid | Granted | Granted; Expected Expiry - 17th Jul 2035 | India |
| KR102260512 B1 | Jeong-yeon Jeong | Inventor | *Bacillus clausii I-52* | - | Granted | Granted; Expected Expiry - 15th Oct 2039 | South Korea |
| US11202807B2 | Univ Wageningen | Academia | *Akkermansia glycaniphilus CBS141023* | Capsule, tablet, powder | Granted | Granted; Expected Expiry - 10th Aug 2037 | Australia, Brazil, Canada, China, Germany, Denmark, Eurasian Patent Organization, Europe, Hong Kong , Israel, India, Japan, South Korea, Mexico, Portugal, USA, PCT* |
| KR102055264B1 | Univ Dong Eui Ind Acad Coop Found | Academia | *Bacillus licheniformis* | Tablet pills, powders, granules, capsules, suspensions, solutions, emulsions, syrups | Granted | Granted; Expected Expiry - 8th May 2037 | South Korea |
| US2021000884 AA | Md Healthcare Inc | Company | *Streptococcus pyogenes* | Powders, granules, sustained-release-type granules, enteric granules, liquids, eye drops, elixirs, emulsions, suspensions, spirits, troches, aromatic water, lemonades, tablets, sustained-release-type tablets, enteric tablets, sublingual tablets, hard capsules, soft capsules, sustained-release-type capsules, enteric capsules, pills, tinctures, soft extracts, dry extracts, fluid extracts, injections, capsules, perfusates, plasters, lotions, pastes, sprays, inhalants, patches, sterile injectable solutions, aerosols, creams, gels, patches, sprays, ointments, plasters, lotions, liniments, pastes, cataplasmas | Under prosecution | Non final action mailed | China, Europe, South Korea, USA, PCT* |
| KR20210148625A | Univ Nat Chonnam Ind Found | Academia | *Lactobacillus saki CVL-001* | Powder, granule, tablet, capsule, gel, hydrogel, suspension, emulsion | Under prosecution | Published application | South Korea, PCT* |
| KR20210058729 A | Catholic Univ Korea Ind Academic Cooperation Foundation | Academia | *Lactobacillus reuteri, Lactobacillus rhamnosus, Lactobacillus Acidophilus, Lactobacillus casei, Lactobacillus casei, Lactobacillus casei, Lactobacillus casei, Lactobacillus casei In the group consisting of Lactobacillus plantarum, Lactobacillus Helveticus, Lactobacillus fermentum, Lactobacillus paracasei and Lactobacillus varicasei, Lactobacillus variobas* | Powders, granules, tablets, capsules, suspensions, emulsions, syrups, aerosols | Under prosecution | Published application | South Korea |
| KR20210058730 A | Catholic Univ Korea Ind Academic Cooperation Foundation | Academia | *Lactobacillus rhamnosus, Lactobacillus reuteri, Lactobacillus Acidophilus, Lactobacillus casei, Lactobacillus plantarum, Lactobacillus Helveticus, Lactobacillus fermentum, Lactobacillus paracasei, Lactobacillus varicasei, Lactobacillus variobas* | Powders, granules, tablets, capsules, suspensions, emulsions, syrups, aerosols | Under prosecution | Published application | South Korea |
| WO12067641 A2 | Nestle Sa | Company | *Enterococcus faecium SF68* | Capsule, tablet, sachet, gravy, drinking water, beverage, yogurt, powder, granule, paste, suspension, chew, morsel, treat, snack, pellet, pill | Under prosecution | Non-entry in european phase | PCT* |
| WO12060884 A1 | Nestle Sa | Company | *Enterococcus faecium SF68* | Capsule, tablet, sachet, gravy, drinking water, beverage, yogurt, powder, granule, paste, suspension, chew, morsel, treat, snack, pellet, pill | Under prosecution | Non-entry in european phase | PCT* |
| WO12039745 A1 | Nestle Sa | Company | *Enterococcus faecium SF68* | Capsule, tablet, sachet, gravy, drinking water, beverage, yogurt, powder, granule, paste, suspension, chew, morsel, treat, snack, pellet, pill | Under prosecution | Non-entry in european phase | PCT* |
| WO21053642 A1 | Sofar S P A | Company | *Bifidobacterium bifidum MIMBb23sg, Lactobacillus paracasei DG®, Lactobacillus paracasei LPC-S01, Lactobacillus paracasei CF3, Lactobacillus rhamnosus GG, Bifidobacterium animalis lactis Bb12* | Tablet, chewable tablet, mouth-soluble tablet, capsule, lozenge, granules, flakes, powder (granules or powder to be dissolved in a liquid or mouth-soluble granules), soft-gel, cream, solution, suspension, dispersion, emulsion, syrup | Under prosecution | Published application | PCT*, Italy |
| US2016263139 AA | Nestle Sa | Company | *Aerococcus, Aspergillus, Bacteroides, Bifidobacterium, Candida, Clostridium, Debaromyces, Enterococcus, Fusobacterium, Lactobacillus, Lactococcus, Leuconostoc, Melissococcus, Micrococcus, Mucor, Oenococcus, Pediococcus, Penicillium, Peptostrepococcus, Pichia, Propionibacterium, Pseudocatenulatum, Rhizopus, Saccharomyces, Staphylococcus, Streptococcus, Torulopsis, Weissella* | Tablets, capsules, liquids, chewables, soft gels, sachets, powders | Under prosecution | Final rejection mailed | Australia, Brazil, Canada, China, Germany, Europe, Spain, Hong Kong , Japan, Turkey, USA, PCT* |
| WO21240399 A1 | Sofar Spa | Company | *Lactobacillus paracasei DG(R) CNCM 1-1572, - Lactobacillus paracasei LPC-S01 DSM 26760, - Bifidobacterium bifidum BbfIBSOI = MIMBb23sg DSM 32708, - Bifidobacterium breve BbIBSOI DSM 33231, - Bifidobacterium breve BblBS02 DSM 33232, - Bifidobacterium animalis subsp. lactis BIIBS01 DSM 33233, - Lactobacillus plantarum LpIBSOI DSM 33234* | Solid, liquid | Under prosecution | Published application | PCT* |
| US2020093872 AA | Univ Rochester | Academia | *Bifidobacterium pseudolongum* | Tablet, capsule, powder, liquid | Under prosecution | Non final action mailed | Canada, Europe, USA, PCT* |
| WO20212528 A1 | Probi Ab | Company | *Lactobacillus paracasei, Lactobacillus plantarum, Lactobacillus rhamnosus, Lactobacillus crispatus, Lactobacillus gasseri, Lactobacillus fermentum, Lactobacillus reuteri, Lactobacillus acidophilus, Lactobacillus helveticus, Lactobacillus casei, Lactobacillus salivarius, and Lactobacillus johnsonii* | Liquid/Solution | Under prosecution | Entry into the national phase | Australia, Brazil, Canada, China, Europe, United Kingdom, South Korea, Mexico, PCT* |
| JP2010173991 A2 | Litanial Bio Science Co Ltd | Company | *Lactobacillus paracasei, Bacillus subtilis natto* | Liquids, suspensions, emulsions, liniments, gels, creams, ointments, patch | Under prosecution | Published application | Japan |
| US2021196769 AA | Univ Brigham Young | Academia | *Lactobacillus rhamnosus, Lactobacillus reuteri, Bacteroides fragilis, Bifidobacterium breve, Bifidobacterium longum, Bifidobacterium pseudocatenulatum, Lactobacillus acidophilus, Lactobacillus brevis, Lactobacillus casei, Lactobacillus salivarius, Lactobacillus lactis, Bifidobacterium bifidum, Bifidobacterium lactis, Bifidobacterium infantis, Lactobacillus plantarum, Lactobacillus delbrueckii, Lactobacillus bulgaricus, Lactococcus cremoris, Enterococcus faecium* | Powders, granules, tablets, pills, capsules | Under prosecution | Non final action mailed | Canada, China, Europe, USA, PCT* |
| KR20210031301 A | Md Healthcare Inc | Company | *Lactobacillus sakei* | Powders, granules, tablets, capsules, suspensions, emulsions, syrups, and creams, gels, patches, sprays, ointments | Under prosecution | Published application | South Korea |
| US2012027737 AA | Chr Hansen A S | Company | *Lactobacillus acidophilus strain LA-5* | Tablets, granules, capsules | Inactive | Abandoned | China, Europe, Hong Kong , USA, PCT* |
| KR20200047097 A | Korea Advanced Inst Sci And Tech | Academia | *Bifidobacterium lactis, Lactobacillus paracasei, Lactobacillus cactus, Lactobacillus plantarum* | Powders, granules, capsules, tablets, aqueous suspensions | Inactive | Rejected | South Korea |
| WO16178493 A1 | Il Dong Bio Science Co Ltd | Company | *Clostridium butyric acid IDCC 5101* | Tablets, capsules, powders, granules, liquids and pills | Under prosecution | Non-entry in european phase | PCT*, South Korea |
| US9314489B2 | 4D Pharma Research Ltd | Company | *Roseburia hominis A2-183* | Tablet, capsule, powder | Granted | Granted; Expected Expiry - 8th Oct 2032 | USA, Europe, PCT*, Australia, Brazil, Canada, China, Cyprus, Germany, Denmark, Spain, United Kingdom, Hong Kong , Croatia, Hungary, India, Japan, Lithuania, Montenegro, Republic of North Macedonia, Mexico, Poland, Portugal, Serbia, Russia, Slovenia, San Marino, Turkey |
| TWI676682 B | Food Ind Res And Dev Inst | Academia | *Bacillus subtilis P12-3C* | Tablets, lipids, capsule, lozenges, drop, transdermal patches, powders, sprays, aerosols | Granted | Granted; Expected Expiry - 31st Aug 2038 | China, Taiwan |
| US10195237B2 | Imagilin Technology Llc | Company | *Pediococcus acidilactici NRRL B-50517* | Tablet, capsule | Granted | Granted; Expected Expiry - 27th Apr 2036 | USA, PCT*, China, Japan |
| FR2962134 B1 | Ingredia SA | Company | *Bifidobacterium longum strain I-3994* | Claims: food | Granted | Granted; Expected Expiry - 5th Jul 2030 | France |
| KR101838281 B1 | Korea Food Res Inst | Academia | *Lactobacillus Cabutus Wikim53* | Tablet | Granted | Granted; Expected Expiry - 12th Dec 2036 | South Korea |
| KR102316396 B1 | Korea Food Res Inst | Academia | *Lactobacillus plantarum WiKim0112* | Tablet, capsule | Granted | Granted; Expected Expiry - 28th Oct 2030 | South Korea |
| US9056123B2 | Gervais Danone Comp | Company | *Lactobacillus rhamnosus DN 116 063 (CNCM I-4271) Streptococcus thermophilus, Lactobacillus bulgaricus* | Yogurt, tablet, capsule, powder, liquid | Granted | Granted; Expected Expiry - 7th Feb 2030 | Brazil, China, Germany, Eurasian Patent Organization, Europe, Spain, India, Poland, Turkey, USA, PCT* |
| KR101862051 B1 | Univ Ind Cooperation Group Kyung Hee Univ | Academia | *Lactobacillus plantarum IM2, Lactobacillus acidophilus IM7, Bifidobacterium longum IM26, Bifidobacterium adolescentis IM38, Streptococcus faecium IM45, Collinsella aerofaciens IM50* | Tablets, pills, powders, granules, capsules | Granted | Granted; Expected Expiry - 16th Jan 2038 | South Korea |
| TWI739424 B | Chambio Co Ltd | Company | *Bacillus species, Lactobacillus species, Bifidobacterium Genus species* | Aseptic powder, tablet, troche, lozenge, pellet, capsule, dispersible powder or granule , solution, suspension, emulsion, syrup, elixirs, syrup, gel | Granted | Granted; Expected Expiry - 12th May 2040 | Taiwan |
| KR101720051B1 | Sunja Food Agricultural Co Ltd | Company | *Lactobacillus rhamnosus GG* | Chewing gum | Granted | Granted; Expected Expiry - 29th Oct 2034 | South Korea |
| KR20190068026 A | Biggert Lactobacillus Co., Ltd. | Company | *Lactobacillus plantarum BK-022* | Powders, granules, tablets, capsules, suspensions, emulsions, syrups, aerosols | Under prosecution | Published application | South Korea |
| WO21112041 A1 | Megmilk Snow Brand Co Ltd | Company | *Lactobacillus delbruecchii Bulgaricus SBT2115,, Lactobacillus herveticus SBT2161, Lactobacillus NITE P-03076, Lactobacillus reuteri SBT10010 , Lactobacillus lactis NITE ABP-03878, Lactococcus lactis SBT0625, Lactococcus lactis SBT2483, Lactococcus lactis SBT1223, Lactococcus lactis SBT 1554, Lactococcus lactis SBT2397, bifidobacteria breve SBT10535, bifidobacteria breve SBT10561, Bifidobacterium breed SBT10535, Bifidobacterium adolescentis SBT10549, Lactobacillus delbrucky bulgaricus SBT0056, Lactobacillus herveticus SBT2192A* | Powder, granule, tablet, capsule, drink | Under prosecution | Published application | PCT* |
| CN113925885 A | Chuang Bai Co Ltd | Company | *Bacillus sp., Streptococcus sp., Lactococcus sp., Atrophic bacteria sp., Balloon sp., Carnivora sp., Enterococcus sp., Lactobacillus sp., Leuconostoc sp. Coccus spp., Pediococcus spp., Tetradococcus spp., Proterococcus spp., Weisseria spp., Bifidobacteria spp., Saccharomyces spp., Kluyveromyces spp., Staphylococcus spp. , Pediococcus sp., Propionibacterium sp* | Sterile powder, troche, tablet, buccal (lozenge), pill (pellet), capsule, dispersible powder, granule, solution, suspension, emulsion, syrup, elixir, slurry, gel, jelly | Under prosecution | Published application | China |
| WO17131402 A1 | Univ Ind Coop Group Of Kyung Hee Univ | Academia | *Lactobacillus plantarum IM2, Lactobacillus acidophilus IM7, Bifidobacterium longum IM26, Bifidobacterium adolescentis IM38, Streptococcus faecium IM45, Collinsella aerofaciens IM50* | Tablets, pills, powders, granules, capsules | Under prosecution | Non-entry in european phase | PCT*, South Korea |
| US2021290736 AA | Lonza Consumer Health Inc | Company | *Firmicutes, Gracilicutes,  Mendocutes, (Bacteroidetes, Actinobacteria, Proteobacteria, Lactobacteria, Bacilli) Verrucomicrobia, Faecalibacteria, Thermophiles, Clostridias, Escherichia coli, Bacteroides fragilis, Bifidobacteria Lactobacteria, (Lactobacteria casei, Bifidobacterium longum ssp infantis, Lactobacteria johnsii, Lactobacteria rhamnosus, Lactobacteria reuteri, Lactobacteria acidophilus, Lactobacteria paracasei, Lactobacillus plantarum, Lactobacillus lundensis) Bacillus coagulans, Bacillus subtilis, Faecalibacteria prausnitzil, Enterococcus faecium, Streptococcus salivarius, Clostridia butyricum, Akkermansia muciniphila Saccharomyces cerevisiae* | Tablets, capsules, gummy chewables, edible films, liquid suspensions, powders, syrups, lozenges, yogurt, fruit juice, beverage matrix | Under prosecution | Published application | Brazil, Canada, Europe, USA, PCT* |
| US2019060415 AA | Lonza Llc | Company | *GPR109a receptor ligand (derived from probiotic/ dietary fiber metabolism)* | Capsules, tablets, gummy chewables, edible films, lozenges, powders, liquid suspensions, syrups | Under prosecution | Final rejection mailed | USA, Europe, PCT*, Brazil, China, Japan |
| WO16072992 A1 | Nwo Stem Cure Llc | Company | *Lactobacillus rhamnosus* | Tablet, lozenge, capsule, packet, stick | Under prosecution | Non-entry in european phase | Canada, China, Japan, South Korea, PCT* |
| US2019046545 AA | Nestle Sa | Company | *Lactobacillus acidophilus, Lactobacillus salivarius, Lactobacillus rhamnosus, Lactobacillus paracasei, Lactobacillus casei, Lactobacillus johnsonii, Lactobacillus plantarum, Lactobacillus fermentum, Lactobacillus lactis, Lactobacillus delbrueckii, Lactobacillus helveticus, Lactobacillus bulgari, Lactococcus lactis, Lactococcus diacetylactis, Lactococcus cremoris, Streptococcus salivarius, Streptococcus thermophilus, Bifidobacterium lactis, Bifidobacterium animalis, Bifidobacterium longum, Bifidobacterium breve, Bifidobacterium infantis, Bifidobacterium adolescentis* | Formula | Under prosecution | Final rejection mailed | Australia, China, Europe, Mexico, Philippines, Russia, USA, PCT* |
| US2021205341 AA | Nestle Sa | Company | *Lactobacillus rhamnosus ATCC 53103, Lactobacillus rhamnosus CGMCC 1.3724, Lactobacillus paracasei CNCM 1-2116, Lactobacillus johnsonii CNCM 1-1225, Streptococcus salivarius DSM 13084 K12, Bifidobacterium lactis CNCM 1-3446 Bb 12, Bifidobacterium longum ATCC BAA-999, Bifidobacterium breve Bb-03, Bifidobacterium breve M-16V, Bifidobacterium infantis Bifantis, Bifidobacterium breve Rosell* | Formula | Under prosecution | Entry into the national phase | Australia, China, Europe, Mexico, Philippines, Russia, USA, PCT* |
| KR20200062064 A | Wonkwang University Hospital | Academia | *Lactobacillus genus strain (Lactobacillus buchneri , Lactobacillus pentosus , Lactobacillus acidophilus , Lactobacillus acidophilus , Lactobacillus plantarum), Bifidobacterium genus strain, Bacillus genus strain (Bacillus coagulans), Leuconostock genus strain, Pediococcus genus strain, Waysella genus strain, Streptococcus genus strain* | Powders, granules, tablets, capsules, suspensions, emulsions, syrups | Inactive | Rejected | South Korea |
| US2013209411 AA | Technische Universitat Munchen | Academia | *Lactocepin derived from L. paracasei (VSL#3) or Lactococcus lactis cremoris SK11* | Tablets, coated tablets, capsules, packages, solutions, suspensions, emulsions, suppositories, pellets, syrups, vaginal suppositories, ointments or creams | Inactive | Abandoned | Germany, Europe, USA, PCT* |
| Sources: WNS Analysis, PatBase, Google Patents | | |  |  |  |  |  |
| Patent Families disclosing joint pain | | |  |  |  |  |  |
| Patent Families disclosing osteoarthritis | | |  |  |  |  |  |
| Patent Families disclosing arthritis | | |  |  |  |  |  |
